# Genome-wide isolation of growth and obesity QTL using mouse speed congenic strains

**DOI:** 10.1186/1471-2164-7-102

**Published:** 2006-05-02

**Authors:** Charles R Farber, Pablo M Corva, Juan F Medrano

**Affiliations:** 1 Department of Animal Science, University of California Davis, One Shields Ave, Davis, CA 95016-8521, USA; 2 Department of Animal Science, University of Mar del Plata, CC 276, 7620 Balcarce, Argentina

## Abstract

**Background:**

*High growth *(*hg*) modifier and background independent quantitative trait loci (QTL) affecting growth, adiposity and carcass composition were previously identified on mouse chromosomes (MMU) 1, 2, 5, 8, 9, 11 and 17. To confirm and further characterize each QTL, two panels of speed congenic strains were developed by introgressing CAST/EiJ (CAST) QTL alleles onto either mutant C57Bl/6J-*hg/hg *(HG) or wild type C57Bl/6J (B6) genetic backgrounds.

**Results:**

The first speed congenic panel was developed by introgressing four overlapping donor regions spanning MMU2 in its entirety onto both HG and B6 backgrounds, for a total of eight strains. Phenotypic characterization of the MMU2 panel confirmed the segregation of multiple growth and obesity QTL and strongly suggested that a subset of these loci modify the effects of the *hg *deletion. The second panel consisted of individual donor regions on an HG background for each QTL on MMU1, 5, 8, 9, 11 and 17. Of the six developed strains, five were successfully characterized and displayed significant differences in growth and/or obesity as compared to controls. All five displayed phenotypes similar to those originally attributed to each QTL, however, novel phenotypes were unmasked in several of the strains including sex-specific effects.

**Conclusion:**

The speed congenic strains developed herein constitute an invaluable genomic resource and provide the foundation to identify the specific nature of genetic variation influencing growth and obesity.

## Background

The use of mouse models has provided valuable insight into the etiology of monogenic syndromes caused by single gene mutations. However, such models do not mimic the genetic complexity of disease traits commonly seen in the human population. Complex traits, such as polygenic growth and obesity are influenced by the small to moderate direct effects of quantitative trait loci (QTL), epistasis between QTL alleles, environmental perturbations and QTL-environment interactions.

To date numerous mouse growth and obesity QTL have been localized [1,2], however, little progress has been made in determining the specific nature of the underlying genetic variants. One resource used to fine map QTL are congenic strains which are designed to convert a complex polygenic trait into one that is mono- or oligogenic. This is accomplished by eliminating segregating genetic variation outside the locus of interest and reducing the environmental variation influencing a trait by characterizing large numbers of genetically identical mice. The Complex Trait Consortium (CTC) considers congenic analysis an excellent method to confirm and subsequently fine map QTL [3].

Traditionally, congenic strains are developed by introgressing QTL alleles from a donor strain, whose boundaries are defined by genetic markers, on the genetic background of a recipient strain via 10 backcrosses [4]. While technically straightforward this is a time consuming endeavor taking over three years to construct a single strain. The speed congenic approach is an alternative to this lengthy process and can reduce the number of required backcrosses from 10 to five [5,6]. This strategy uses marker-assisted selection to identify male mice inheriting fewer donor alleles, than expected on average, during each backcross. Numerous traditionally developed and speed congenics have been used to successfully isolate mouse QTL for a wide array of traits, including growth and obesity [7-13].

The C57Bl/6J-*hg/hg *(HG) mouse is a model of systemic overgrowth resulting from a spontaneous deletion on MMU10, which eliminates expression of the *Socs2 *(suppressor of cytokine signaling 2) gene [14-16]. The role of *Socs2 *in the HG phenotype was confirmed by an independently engineered *Socs2*^-/- ^knockout mouse which shared a number of phenotypes in common with HG, including gigantism [17].

QTL which alter the phenotypic effects of another locus are referred to as modifier QTL [18]. Modifier QTL have been mapped for numerous traits and in these studies the modified locus is typically a known gene containing a spontaneously arisen or engineered mutation with major phenotypic effects, such as *hg *[11,19-21]. Epistasis forms the basis of these interactions, implying that the known gene and its modifiers are members of the same biochemical or metabolic pathway. Different genetic backgrounds have been shown to modify the growth-enhancing effects of *hg *[15,22]. Since the primary function of *Socs2 *is to negatively regulate growth hormone (*Gh*) [23], it is likely these background effects are the result of polymorphism influencing interactions between members of the *Gh *signaling pathway. Thus, identification of *hg *modifiers has the potential to uncover novel members of metabolically important pathways or previously unknown convergences between pathways.

As an initial step, growth and carcass composition QTL were identified in a cross between CAST/EiJ (CAST) and HG [24]. In the F_2_, mice homozygous for the *hg *deletion (*hg/hg*) and wild type (+/+) were assayed and *hg *modifier QTL were defined as those either absent in +/+ mice and segregating in *hg/hg *or loci with altered gene action dependent on background. Four *hg *modifier QTL (*Wg2 *on MMU2, *Carfhg2 *on MMU9, *Carp2 *on MMU11 and *Feml3 *on MMU17) were identified, along with 12 additional QTL (*Q1Ucd1 *on MMU1 (which only reached a suggestive level of statistical significance), *Wg1, Carp1, Cara1 *and *Feml1 *on MMU2, *Carfhg1 *on MMU5, *Wg3 *on MMU8, *Feml2 *on MMU9, *Wg4 *and *Cara2 *on MMU11 and *Carp3 *and *Cara3 *on MMU17) whose expression was independent of the *hg *locus.

In the current study we have developed speed congenic strains to isolate all the aforementioned QTL. Single strains on an HG background were created for each chromosomal region outside of MMU2, while a comprehensive panel of overlapping strains with identical donor regions on both B6 and HG backgrounds were developed for MMU2. The MMU2 panel was developed to confirm the presence of multiple QTL and test for QTL-*hg *interactions. Additionally, based on the knowledge of potential QTL-*hg *interactions, genes involved in *Gh *signaling, whose genomic location overlapped *hg *modifier QTL on MMU2, 9, 11 and 17, were sequenced.

## Results

### Speed congenic strain development

**Figure 1:**
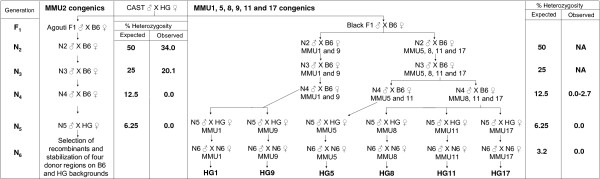
**Outline of the speed congenic approach used to capture genome-wide growth and obesity QTL**. Details of the congenic matings are outlined in "Methods". The matings used to construct the MMU2 speed congenic panel are listed on the left of the figure, along with the expected and observed % heterozygosity at each generation. % heterozygosity is defined as the number of markers heterozygous B6 (or HG)/CAST as a percent of the total number of markers typed (N = 79). The right side of the illustration lists the matings used to construct the MMU1, 5, 8, 9, 11 and 17 speed congenic strains, along with the expected and observed % heterozygosity at each generation.

Two speed congenic panels were created, the first comprehensively dissected MMU2 while the second isolated QTL on MMU1, 5, 8, 9, 11 and 17 (Table 1). The MMU2 panel consisted of eight congenic strains developed by introgressing four overlapping donor regions onto both B6 and HG genetic backgrounds (Table 1). Single donor regions bred onto an HG background were created to isolate the remaining QTL on MMU1, 5, 8, 9, 11 and 17. Implementation of a speed congenic approach using marker-assisted breeding with 79 genome-wide microsatellite markers accelerated production of all strains [5,6] (Additional File 1 and Figure 1). Strain abbreviations and genomic region isolated by each strain are listed in Table 1. In addition to both congenic panels, two control strains homozygous B6 (B6.CASTC; B6C) or HG (HG.CASTC; HGC) for all genome-wide markers genotyped were developed from the same cross (see Methods) and served as the basis for strain comparisons. After stabilizing each congenic, 12 of 14 were phenotypically characterized for growth and adiposity. The HG2D and HG5 strains were not characterized due to reproductive problems. The recombinant end points for all strains were refined using microsatellite markers flanking each donor region (Additional File 2).

### MMU2 speed congenic strain characterization

**Figure 2:**
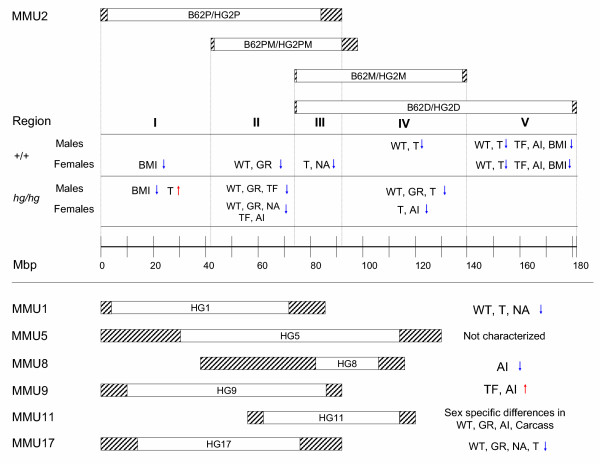
**Genome-wide speed congenic strains and summary of QTL effects**. White bars indicate the boundaries of CAST donor regions and hatched bars indicate intervals of unknown genotype. The minimum physical intervals (Mbp) of each donor region are listed in Table 1. To provide a general summary of the results, MMU2 is divided into five chromosomal regions (I, II, III, IV and V). Phenotypic differences for male and female (+/+ and *hg/hg*) mice relative to the appropriate controls are listed. For congenics outside of MMU2, a general summary of QTL effects is listed to the right of each donor region. Abbreviations: WT, body weight; GR, growth rate; T, tail length; NA, nasal-anal body length; TF, total weight of four fat pads; AI, adiposity index and BMI, body mass index.

Due to the overlapping nature of the MMU2 congenics, five distinct chromosomal regions (Regions I–V) were queried for the presence of QTL (Figure 2). As a separate analysis we also tested for interactions between each donor region and the two distinct genetic backgrounds. The following sections describe the phenotypes for each B6.CAST and HG.CAST speed congenic strain as well as the results of the interaction analysis.

#### B6.CAST MMU2 speed congenic strains

**Figure 3:**
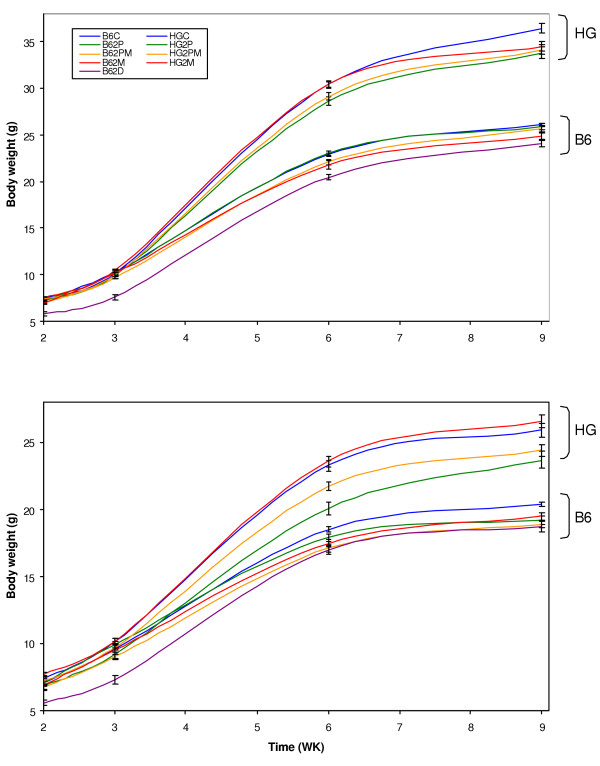
**Growth curves for MMU2 B6.CAST and HG.CAST speed congenic strains**. Body weight LSMEANS ± SEM are plotted as a function of time (weeks). Top plot, growth curves in male B6 (+/+) and HG (*hg/hg*) speed congenic strains; bottom plot, growth curves in female B6 (+/+) and HG (*hg/hg*) speed congenic strains.

The B62D strain exhibited the largest (P < 0.0001) decreases in body weight of any B6.CAST congenic (Additional File 3). Both sexes had reductions in weight at 2 (2WK), 3 (3WK), 6 (6WK) and 9 (9WK) weeks of age compared to control B6C mice (Figure 3). Despite large decreases in body weight, no differences in growth rates (G26, weight gain from 2 to 6 weeks and G29, weight gain from 2 to 9 weeks) were observed (Additional File 3). Therefore, the B62D unique region (Region V) harbors either an early-growth QTL or maternal genotype effect which produces a distinct decrease in body weight prior to 2WK. These effects are evident in the growth curves for both sexes of each strain (Figure 3). Additionally, B62D mice displayed significantly shorter tail lengths despite no difference in naso-anal length (NA) (Table 2).

In contrast, B62M mice displayed small decreases in body weights and growth (male 9WK was significant, female 6WK and 9WK were suggestive at P < 0.05) and significant decreases in NA (male P = 0.0152) and tail length (Additional File 3 and Table 2). The decrease in NA in B62M mice is surprising since the 2M donor region is entirely nested within the 2D donor region and B62D mice showed no difference in NA (Figure 2). These data indicate the presence of three QTL with the 2D unique region. Two of which are disjoined by the B62M and B62D strains and additively decrease body weight and tail length, but not NA. The third QTL, located within the B62D unique region (Region V), increases NA canceling the effects of the B62M QTL (Region IV) decreasing NA.

B62PM females had decreases in all growth-related traits except 2WK and 3WK, while no differences were seen in males (Additional File 3). Similarly, only B62P females and not males showed significant decreases in 9WK, G26 and G29 (Additional File 3), indicating these two strains share a female specific growth QTL (Region II).

In the original genome scan body fat percentage as determined by chemical compositional analysis was not linked to markers on MMU2 [24]. However, since numerous obesity QTL have been found on MMU2 [2], some of which were discovered in B6 by CAST crosses [9,25], we chose to measure dissected fat pad weights as a more sensitive measure of adiposity.

Table 2 lists the weights of gonadal (GFP), femoral (FFP), mesenteric (MFP) and retroperitoneal (RFP) fat pads, along with total fat pad weight (sum of the four fat pads), adiposity index (AI = TF/weight at sacrifice (WSAC)) and body mass index (BMI = WSAC/NA^2 ^*100) for each MMU2 congenic strain. Similar to large differences in body weight the B62D strain displayed highly significant decreases in adiposity. B62D was the only strain where both sexes had significant decreases in TF, AI and BMI. Interestingly, no difference in adiposity was seen in B62M mice. This discordance indicates the leanness promoting QTL is located in the B62D unique region (Region V). Only minor differences in various fat pad weights were observed for the B62P and B62PM strains (Table 2).

#### HG.CAST MMU2 speed congenic panel

HG2P and HG2PM mice of both sexes displayed significant decreases in post-weaning body weights and growth rates (Additional Table 3). In addition, the HG2M strain displayed highly significant decreases in length, similar to B62M mice (Table 2). These results indicate QTL within Region II shared between the HG2P and HG2PM strains decrease body size, weight gain and NA (only in females), while a QTL in the HG2M region significantly decreases tail length.

Similar to growth traits a general decrease in GFP, MFP, RFP, TF and AI were seen in HG2P and HG2PM mice (Region II) (Table 2). These differences in adiposity are likely pleiotropic effects of the growth QTL shared between these two strains. Surprisingly, HG2M mice displayed striking sex differences in adiposity. Males exhibited an increase in AI (P = 0.0256) with a decrease in BMI (P = 0.0462) and the exact opposite was seen in females (AI, P < 0.0001; BMI, P = 0.0425) (Table 2). Therefore, the obesity QTL in the HG2M unique region (Region IV) is profoundly impacted by the presence of *hg*.

#### Confirmation of QTL-hg interactions

An important feature of our experimental design was the ability to test for interactions between QTL in each MMU2 donor region and genotype at the HG locus, since identical donor regions were introgressed on two genetic backgrounds, B6 (+/+) and HG (*hg/hg*). Significant interactions between donor region and HG genotype were viewed as strong evidence that *hg *modifier QTL reside within that unique genomic region.

**Figure 4:**
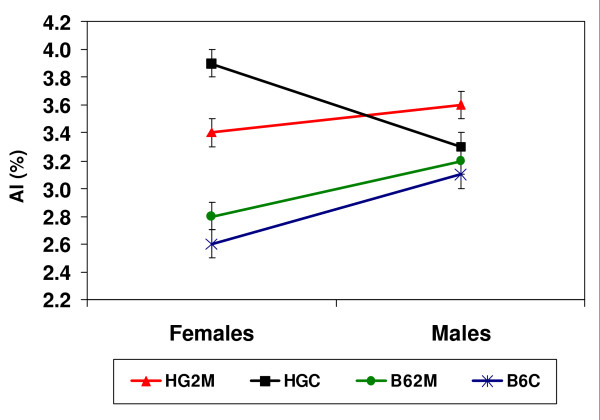
**Significant three-way donor region by HG genotype by sex interaction for AI in the 2M donor region**. AI plot illustrates the effect of the *hg *deletion on altering fat deposition between the sexes. The interaction is significant at P = 0.0004.

In the original linkage analysis the mode of gene action and peak location of *Wg2 *were dependent on the presence of *hg *[24] (Table 1). In the present study, only homozygous congenic mice were characterized, thus, the overdominant effects of *Wg2 *were not tested. However, since its original peak location differed dependent on background we hypothesized that *Wg2 *represents a set of linked QTL between 74.9 and 181.8 Mbp within the 2M and 2D donor regions (Region III–V), some of which interact with *hg*. Following this logic we expected one or both of these strains would exhibit donor region by HG genotype interactions. Unfortunately, we were unable to characterize HG2D mice. However, as noted above, strong sex-specific effects were seen in HG2M mice. Significant 2M donor region by HG genotype by sex three-way interactions for AI (P = 0.0004; Figure 4) and TF were identified (Table 3). The basis of these interactions was a decrease in HG2M female and an increase in HG2M male adiposity, with no differences in fat accumulation across B62M sexes. Significant 2PM donor region by HG genotype two-way and 2PM donor region by HG genotype by sex three-way interactions were also seen for TF and AI (Table 3). In addition, significant 2P donor region × HG genotype interactions were observed for all traits listed, although some traits did reached significance at the critical P < 0.0071 (Table 3). The basis for each interaction was a decrease in phenotype in HG2P compared to HGC and no difference between B62P and B6C. Together these data confirm that MMU2 QTL modify the effects of the *hg *deletion.

### Speed congenic strains for QTL on MMU1, 8, 9, 11 and 17

#### HG1

*Q1Ucd1 *affecting G26 was the least significant of any QTL identified in the original intercross and only reached a suggestive level of significance (LOD = 2.46, P < 0.05) [26]. In accordance, HG1 mice had small differences in all growth and length traits. NA in both sexes and 2WK, 3WK, 6WK and tail length in females was significant at a critical P < 0.005 (Table 4). The only difference in adiposity was a significant decrease in male MFP (Table 5). The successful capture and confirmation of the small effect *Q1Ucd1-w26 *QTL adds support to pursuing QTL identified at suggestive levels of statistical confidence from genome scans.

#### HG8

Markers on MMU8 were linked with G29 in the original intercross [24]. This QTL was small, only accounting for 4.3% of the phenotypic variance. Interestingly, in HG8 mice no differences in growth were detected; however, they were significantly leaner than controls (Table 5). In males a general decrease in fat pad mass, TF and AI was detected; however, only MFP was significant at a critical P < 0.005. Male AI was nearly significant at a nominal P = 0.0082. Larger decreases in fat mass were seen in HG8 females, with significant decreases detected for GFP, MFP and AI. Additionally, RFP and TF were nearly significant with nominal P values of 0.0208 and 0.0153, respectively. HG8 mice represent a lean congenic model in which CAST alleles protect against fat accumulation.

#### HG9

CAST alleles in the proximal region of MMU9 were previously found to be linked with an increase in body fat percentage and a decrease in femur length [24]. The effects appeared to be the result of two linked QTL, *Carfhg2 *at 10 cM and *Feml2 *at 20 cM, instead of one pleiotropically impacting both traits (Table 1). In support of this, the increase in fat was dependent on *hg*; however, differences in femur length were not [24].

In confirmation of *Carfhg2 *all measures of adiposity in HG9 mice were increased relative to control mice; except MFP in both sexes and BMI in males (Table 5). In males, GFP, RFP, FFP and AI were increased by 29.0%, 51.2%, 42.9% and 30.0%, respectively. In females these increases were much larger. The same measurements were increased by 68.8%, 99.4%, 69.7% and 57.3%, relative to HGC females. A 2.9% and 3.6% reduction in male and female NA, respectively, was also detected. This was expected since NA was previously found to be an excellent indicator of femur length (R^2 ^= 0.88) in HG mice [27] and as noted above this region contains the *Feml2 *QTL [24]. These data confirm the effects of *Carfhg2 *on adiposity and *Feml2 *on length and indicate the isolated effects of *Carfhg2 *are more significant than originally observed.

#### HG11

In the original intercross CAST alleles near 50 cM on MMU11 were linked with a reduction in G29, carcass ash (ASH) and carcass protein (PROT) [24]. Therefore, we measured each carcass component using chemical compositional analysis in the HGC, HG11 and HG17 strains. Originally, *Carp2 *was dependent on the presence of *hg *and no significant sex effects were detected [24]. In contrast to those results, HG11 mice displayed significant differences in growth and obesity between the sexes. In general, males demonstrated a 5% increase and females a 5% decrease in all growth-related traits (Table 4). Most of these differences, however, were not significant at a critical P < 0.005, although almost all were at a nominal P < 0.05. Following the same trend, males displayed an increase in tail length and a small suggestive increase in NA (P = 0.06), while females displayed significant decreases in NA. In support of the sex-specific QTL effects strain by sex interactions (P < 0.04) were seen for the following growth traits: 3WK, 6WK, 9WK, G26, G29 and NA (data not shown). The basis of each interaction was an increase in the males and a decrease in females.

In our analysis of the HGC, HG11 and HG17 strains, concordant results were observed measuring adiposity using chemical lipid extraction and weighing fat pad mass. The phenotypic correlation between carcass fat (FAT) as a percent of the empty carcass weight (ECW; weight of total body (including organs) minus the head and gastrointestinal tract) (%FAT) determined by chemical analysis and AI, using data from all three lines (HGC, HG11 and HG17), was 0.92. These data suggest that individual fat pad dissection is an excellent proxy for measuring whole body adiposity and provides a much more sensitive technique to measure fat accumulation in specific body regions.

HG11 females displayed slight increases in all fat pads (except MFP) and AI, although, only the 16.5% increase in AI was significant (Table 5). In contrast, HG11 males did not show a difference in fat mass leading to a significant strain by sex interaction (P = 0.008) for AI (data not shown). Identical to the results obtained using fat pad weights; female HG11 carcasses displayed an increase in %FAT (Table 6).

HG11 male carcasses had higher levels carcass ash (ASH) as a percent of ECW (%ASH) and female carcasses displayed lower levels of H_2_O and ASH (Table 6). Strains by sex interactions (P < 0.005) were identified for H_2_O, %H_2_O, %FAT, ASH and PROT (data not shown). If the aggregate phenotype in HG11 is due to a single locus, its function may be to disrupt energy partitioning by decreasing the deposition of lean tissue and increasing lipid accumulation in females, while having the opposite action in males. However, it may also be due to distinct sex-specific QTL with opposing action.

#### HG17

In the original intercross CAST alleles at MMU17 markers were associated with decreases in femur length, ASH and PROT [24] and similar results were seen in HG17 mice. Interestingly, both sexes were heavier at 2WK (males, P = 0.0045; females, P = 0.0342), but lighter at 9WK, leading to substantially lower G26 and G29 (Table 4). In general growth differences ranged between 5% and 15% lower in congenic mice.

Both sexes had significant reductions in length traits relative to control mice. NA was reduced by 2.7% (P = 0.0186) and 3.4% in males and females, respectively (Table 4). In addition, tail was reduced by approximately 6% in both sexes (Table 4).

In general, HG17 mice displayed increases in all fat pads (except MFP), TF and AI (Table 5). However, none of these differences were significant at critical P < 0.005. In addition, HG17 male carcasses possessed lower levels of H_2_O and ASH, while female carcasses contained lower levels of % H_2_O, ASH, %ASH and %PROT (Table 6).

### MMU2, 9, 11 and 17 hg modifier candidate gene sequencing

**Figure 5:**
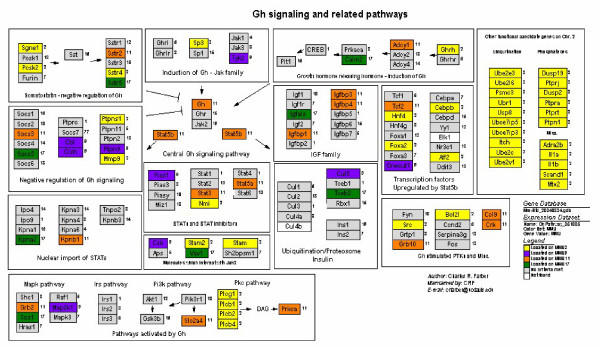
**GenMAPP growth hormone (*Gh*) signaling pathway**. Genes known to be or potentially involved in *Gh *signaling were mined from primary literature, reviews and book chapters. Genes are organized broadly based on functionality. MMU genomic position for each gene is to the right of each symbol and genes located on MMU2, 9, 11 and 17 are highlighted in yellow, purple, orange and green, respectively. Genes located on MMUX are labeled in white. Forty-four of the genes overlapping QTL intervals were selected for sequencing in the CAST strain.

The HG phenotype is due to the deletion of *Socs2*, therefore we reasoned that QTL on MMU2, 9, 11 and 17 interacting with *hg *possibly represent variation within genes participating in various aspects of *Gh *function. To select candidate genes for sequencing we identified genes known to be or potentially involved in *Gh *signaling, are responsive to *Gh *or that propagate downstream *Gh *functions. Forty-four *hg *modifier candidate genes were identified from primary literature, reviews and book chapters and coordinated using the GenMAPP (Gene MicroArray Pathway Profiler) pathway building software (Figure 5). The coding region of each gene was sequenced from the CAST strain and compared to the publicly available B6 sequence to identify polymorphisms (Additional File 4). A total of 94.492 kbp was sequenced (75.378 kbp CDS and 19.114 Kbp 5' and 3' untranslated region (UTR)), representing 25,083 amino acids. Comparison with the public B6 assembly (May 2004 University of California, Santa Cruz (UCSC [28]) mm5 genome assembly, National Center for Biotechnology Information (NCBI) Build 33) identified 307 polymorphisms between CAST and B6. Of these, 295 were single nucleotide polymorphisms (SNP) and 12 were insertions or deletions in CAST (Additional File 4). All 12 insertions or deletions were located within 5' and 3' UTRs. Fifty-six nonsynonomous SNP (nsSNP) were identified in 14 different genes (Additional File 4). PolyPhen [29] and SIFT [30] are software programs designed to identify nsSNP which potentially alter protein function by evaluating evolutionary conservation at specific amino acid residues using a multiple sequence alignment of protein sequences homologous to the query. When applied to our data set PolyPhen, SIFT or both programs predicted that 15 of the 56 nsSNP in 9 different genes would possibly alter protein function (Table 7).

## Discussion

Speed congenic strains provide a powerful approach to confirm and physically confine QTL within intervals defined by molecular markers. In the current study, approximately 20% of the CAST genome harboring all previously detected growth and carcass composition QTL was isolated on an HG or B6 background through the development of 14 speed congenic strains. Two distinct speed congenic panels were developed, the first provided a comprehensive isolation of all MMU2 QTL between the B6, HG and CAST strains and the second targeted all QTL outside of MMU2. Each successfully characterized strain exhibited phenotypic differences relative to control mice. These strains represent important resources and provide the genetic resource to positionally clone numerous quantitative trait genes.

One criticism of the speed congenic approach is the potential for QTL to reside among unwanted donor alleles not eliminated during backcrossing. In this case differences between the congenic and control strains would be due in part or whole to these contaminating alleles. We took several precautions to reduce the probability of this occurrence. First, our control strains were developed from mice undergoing the same selection as all of the other congenics. Therefore, it is possible that any unwanted QTL or mutations arising during congenic construction are shared between all strains. More importantly, we have knowledge of all large previously detected growth and obesity QTL in the current cross [24]. Using this information we increased the density of markers in each QTL region (MMU1, 2, 5, 8, 9, 11 and 17; Table 1 and Additional File 1), ensuring the absence of CAST alleles at each of these QTL. This approach, termed "QTL-Marker-Assisted Counter Selection" or QMACS, has been previously used to characterize QTL for hypnotic sensitivity to ethanol [31]. In that study, only markers flanking QTL were typed, not genome-wide markers. In contrast, we selected not only against known QTL, we also screened for genome wide heterozygosity increasing the probability that effects observed are due to genetic variation within each donor region.

Although great effort was put forth to eliminate non-donor region direct genetic effects, other factors such as maternal genotype (maternal genotype for each congenic versus control dams differed) and environmental effects could confound our results. Maternal genotype effects on growth and obesity have been observed in a number of mouse crosses [8,32-35] and their existence in the current cross cannot be discounted. However, our congenics provide the ideal foundation genomic resource to test for the influence of any of these possible effects. Future fine mapping experiments can be designed to randomize the influences of any contaminating donor alleles and environmental differences, as well as test for maternal genotype effects.

MMU2 is a hotspot for growth and obesity QTL. Over 30 QTL have been identified in various experiments [1,2]. Several previously reported or novel MMU2 QTL have been isolated and characterized using congenic strains [7,9-11,36]. Our findings are no different and indicate that MMU2 is highly complex with regards to genes affecting growth and obesity. The overlapping nature of our MMU2 strains allowed us to parse the chromosome into five regions (Regions I–V) (Figure 2). The data support the presence of at least one QTL in each of the five regions (Figure 2). Each of the five pleiotropically impact both growth and obesity, although to varying degrees.

In addition to the large number of MMU2 QTL, the presence of *hg *adds complexity by either eliciting interactions with the same QTL or by inducing the expression of novel QTL. The 2P unique region (Region I) contains an *hg *modifier with large effects on growth and smaller effects on obesity (Table 3). In contrast, interactions between 2PM/2M QTL and *hg *primarily affect fat deposition (Table 3). As illustrated in Figure 4 the 2M donor region exhibits strong sex effects on the rate of lipid storage, dependent on background. In control mice, HGC females have a higher AI than males, while the opposite is seen in B6C mice. Interestingly, the 2M *hg *modifier QTL abolished the *hg *induced sexual dimorphism in adiposity (Figure 4). Although these results provide insight into the nature of *hg *modifier QTL, it should be noted, that the actual number and precise location of loci is still unclear.

In addition to MMU2, three other congenics (HG9, HG11 and HG17) captured *hg *modifier QTL. In classical terms, they are QTL which modify the expressivity of growth and obesity in HG mice [18]. These QTL are novel since they represent epistasis between *hg *and the modifier gene. QTL-*hg *epistasis implies that *Socs2 *and the *hg *modifiers are in the same biological pathway. Therefore, these QTL are likely due to polymorphism in genes interacting with *Gh*, responsive to *Gh *or which in some way modulate *Gh *function. This information will significantly aid QTL cloning by providing another filter to screen candidates. Cloning these QTL has major implications to improve our understanding of *Gh *and its regulation of growth and adiposity and in the administration of human *Gh *therapeutics.

HG1 mice displayed differences only in growth traits. Originally, *Q1Ucd1 *had small effects and these results illustrate the power of congenic strain analysis to isolate small effect QTL, although it is likely this QTL represents the lower boundary of detection using only 20–30 congenic mice. The most notable candidate genes located within the HG1 interval are the signal transducer and activator of transcription 1 (*Stat1*) and *Stat4*.

Two of the congenics had major alterations in the deposition of adipose tissue, HG8 and HG9. The HG8 donor region promotes leanness. Congenic females displayed a reduction of over 25% in AI. In contrast, the HG9 strain is an obese mouse model. The strain is quite novel for two reasons. First, the effect size is large; AI was 57% higher in HG9 females and 30% higher in males. Secondly, it is dependent on *hg *for its expression. The HG9 strain represents a major epistasis-based obese mouse model and promises to aid in the understanding of obesity and specifically the modulation of adipose tissue deposition by *Gh*. Studies are currently underway to identify the causative mutation and to characterize the effects of age and diet on obesity in this strain as well as testing for other physiological consequences such as alterations in food intake and insulin sensitivity.

The HG11 strain is of particular interest for a number of reasons. First, HG11 congenic mice demonstrated significant strain by sex interactions for a number of traits. Males were generally larger, faster growing and longer and the converse was seen in females. The confounding effects of sex are likely the reason for the discrepancies between the congenic and genome scan results, where both sexes were analyzed together. Secondly, *Carp2 *was found to interact with *hg *and MMU11 is saturated with genes involved in the central *Gh *intracellular signaling pathway, such as *Gh*, *Stat5b *and *Stat5a*. Thirdly, MMU11 growth QTL overlapping HG11 have been identified in a number of crosses using different mouse strains [37-41]. Given the well documented sexually dimorphic nature of *Gh *secretion and *Gh *induced gene expression [42,43], it is probable that the underlying mutation in the HG11 congenic may reside in a gene enhancing *Gh *induced sex-specific effects. The structural *Gh *gene itself and *Stat5b *are excellent candidates. The potential role of *Gh *would include polymorphism that alters protein function in the absence of *Socs2 *or that causes transcriptional deregulation of *Gh*. Additionally, functional variation in *Stat5b *may explain the sex-specific phenotypes in HG11 mice since it is the primary transcription factor responsible for *Gh *induced sex-specific liver gene expression [44]. Future studies aimed at identifying the HG11 QTG, will certainly include a thorough characterization of *Gh *and *Stat5b *sequence and expression patterns in congenic mice.

The *hg *modifier QTL located within the HG17 strain had large effects on growth, body length and carcass components. The most intriguing candidate gene located in the congenic is *Sstr5*. Somatostatin is a potent inhibitor of *Gh *and *Sstr5 *is one of five receptors which mediate these effects [45,46]. Ubiquitous and pancreatic beta cell specific knockout of *Sstr5 *leads to alterations in insulin secretion and glucose homeostasis [47,48].

We sequenced candidate *hg *modifier genes to complement the characterization of the speed congenics on MMU2, 9, 11 and 17. A limited number of studies have identified variation within CAST coding sequence; so sequencing candidates gave us the opportunity to estimate the SNP frequency in coding sequence relative to B6. This information will be vital to future fine mapping studies, which will include gene expression analysis. The high SNP frequency (0.312%; 295 SNP in 94.492 kbp) in CAST genes may lead to a higher rate of false positives using DNA microarrays or quantitative real-time PCR assays, most of which are based on B6 sequence. Therefore, these sequence data can be used to guide the development of gene expression assays to confirm differential expression for candidates and suggests that genes identified by downstream experiments should also be sequenced.

More importantly, sequencing *hg *modifier candidates allowed us to identify nonsynonomous polymorphism, which may underlie QTL. SIFT and/or PolyPhen predicted alterations in protein function for 15 of the 56 total nsSNP (27%) in nine of the 44 total genes (20%) (Table 7). It has been shown that predicting function based on evolutionary conservation using programs such as SIFT and PolyPhen is very accurate [49]. Four of the nine genes (*Ubr1*, *Ptpns1, Ubce7ip5 *and *Mmp9*; all of which are on MMU2) are of particular interest. It has been suggested the *Socs2 *may rely on ubiquitination dependent proteasomal degradation to inhibit *Gh *signaling [23]. *Ubr1 *and *Ubce7ip5 *are both involved in protein ubiquitination and *Ubr1 *knockout mice show an 20% decrease in body weight partly due to a reduction in adipose tissue [50,51]. *Ptpns1 *knockout mice displayed a 10% reduction in body weight [52] and *Mmp9 *knockout mice show a reduction in size and drastically reduced bone length [53]. All of these genes are excellent functional and positional *hg *modifier candidate genes and this information can be incorporated into future fine mapping studies.

## Conclusion

The speed congenic strains developed herein confirm previously identified genome-wide QTL affecting growth, obesity and carcass composition. Our novel congenic development approach on MMU2 provided confirmation of QTL-*hg *interactions, which will significantly aid in future fine mapping experiments. The identification of the molecular mediators of QTL-*hg *epistasis as well as the QTG for the remaining congenics will provide essential information on the molecular genetic architecture of growth and obesity.

## Methods

### Mouse strains and husbandry

CAST/EiJ (stock #000928) mice were purchased from the Jackson Laboratory. The *hg *mutation was originally identified in a selected outcross line [22] and has been introgressed onto a B6 background via nine backcrosses to create the HG strain. HG mice used in this experiment were from the 17^th ^or later generation of inbreeding. Mice were housed in polycarbonate cages under controlled conditions of temperature (21°C ± 2°C), humidity (40–70%) and lighting (14 h light, 10 h dark, lights on at 7 AM), and managed according to the guidelines of the American Association for Accreditation of Laboratory Animal Care (AAALAC).

### Genotyping

DNA for genotyping was isolated from 1.0–2.0 mm tail clips by digesting with Proteinase K (Fisher) at 55°C in a buffer composed of 0.45% NP40 (Sigma), 0.45% Tween 20 (Fisher) and 1X PCR buffer (Promega). The product of this digestion was diluted (1:10) in sterile H_2_O and used for genotyping without further purification. Microsatellite genotyping was performed using standard PCR and gel electrophoresis protocols. Reaction conditions for each marker are listed in Additional File 1 and 2.

MMU2 congenic mice were genotyped for *hg *using a two primer genotyping assay. One primer set (HG-F, ctcctgtctgggctgtgag and HG-R, caaaggcagaagtggggtaa) spanned the *hg *deletion producing a 447 bp product in *hg/hg *and *+/hg *mice. The other set (CRADD3a.F, gtccatcagcattcctgaaa and CRADD3.R, tgtccagcaacagcattgtc) amplified a 232 bp *Cradd *amplicon (located within the *hg *deletion) in *+/+ *and *+/hg *mice [54]. The PCR annealing temperature was 55°C and the MgCl_2 _concentration was 1.5 mM.

### Development of B6.CAST and HG.CAST MMU2 speed congenic strains

All speed congenic strains were developed starting with an initial cross between a CAST male and HG females (Figure 1) [5,6]. Male F_1 _mice were then backcrossed to HG females. All agouti (the dominant *nonagouti *(*a*) locus is located at 154.8 Mbp on MMU2) N_2 _males were genotyped for 79 microsatellite markers (Additional File 1). These markers were evenly spaced across the genome, except in regions previously identified as harboring QTL [1], which were more densely screened. The "best" N_2 _agouti male with the lowest level of genome-wide unwanted heterozygosity while maintaining CAST alleles for all MMU2 markers was selected for breeding. This selection scheme was used at each generation until a N_4 _male was identified as homozygous HG for all markers typed outside MMU2. After an additional backcross to HG females, recombinant males were identified providing the foundation for the four overlapping donor regions. Selected recombinant males were then backcrossed to both B6 and HG females to create strains, which were B6 (+/+) or HG (*hg/hg*) and heterozygous congenic. These mice were intermated to produce homozygous founders for each strain. This novel breeding scheme created four identical founder congenics on two backgrounds B6 (+/+) and HG (*hg/hg*), which formed the basis for our examination of interactions caused by the presence of the *hg *deletion. MMU2 speed congenic strains were maintained through brother-sister mating. Once each congenic was stabilized, 20 additional microsatellite markers were used to refine the position of each congenic recombinant end point (Additional File 2).

### Development of HG.CAST speed congenic strains for MMU1, 5, 8, 9, 11 and 17

All black N_2 _males from the first two crosses described above were genotyped for 12 markers (*D1Mit432*, -*480*, *D5Mit353*, -*311*, *D9Mit60*, -*262*, *D11Mit5*, -*67*, *D8Mit234*, -*211 *and *D17Mit28 *and -*142*), two spanning each of the six QTL harboring regions (MMU1, 5, 8, 9, 11 and 17; Table 1 and Additional File 1). Markers were selected to capture, at a minimum, the 2-LOD support interval. Two N_2 _males were selected to propagate the N_3 _generation; one heterozygous for QTL on MMU1 and 9 and the other heterozygous for QTL on MMU5, 8, 11 and 17 (Figure 1). Both males were homozygous for HG alleles at all other known QTL. These males were backcrossed to HG females and two of the resulting N_3 _males inheriting the same sets of QTL as their sire were selected for breeding. These males were subsequently backcrossed to HG females and three N_4 _males were identified heterozygous for the following regions: 1) MMU1 and 9; 2) MMU5 and 11; 3) MMU8, 11 and 17 (Figure 1). Starting at N_4 _and continuing through N_6_, the "best" male with the lowest percent of unwanted donor alleles was selected after performing a genome scan using the remaining 67 genome-wide markers (79 total markers minus the 12 markers genotyped in the first two backcrosses spanning the know QTL intervals). At N_5 _a distinct strain was created for each of the six individual donor regions and heterozygous mice were intermated (Figure 1). Homozygous HG.CAST speed congenic strains were maintained through brother-sister mating. Once each congenic was stabilized, 19 additional microsatellite markers were used to refine the position of each congenic recombinant end point (Additional File 2).

### Development of B6.CASTC and HG.CASTC control strains

HG is a strain in which the *hg *deletion has been introgressed onto a B6 background, therefore the only genetic differences between the strains would be the *hg *locus, tightly linked alleles from the outbred strain on which *hg *arose and contaminating alleles remaining after the nine backcrosses and fixed during inbreeding. Instead of using parental B6 and HG strains as controls for phenotypic comparisons with each speed congenic, we choose to develop independent control strains originating from the same cross as the congenic panels. Separate B6.CAST control (B6C) and HG.CAST control (HGC) strains were developed using mice from the MMU2 experiment. Mice from the last backcross inheriting only B6 or HG MMU2 alleles at markers spanning MMU2 were intermated to serve as the basis for each control. Both control strains were subsequently maintained through brother-sister mating.

The control strains were coisogenic with the parental B6 or HG strain with the exception of mutations that arose during congenic construction and a small percentage of contaminating donor alleles missed after 6 backcrosses. Therefore, since the congenics and controls were developed through the same selection scheme and possibly share common contaminating regions, the B6C and HGC strains are the most ideal control to compare each congenic.

### Phenotypic characterization

Trait data were collected on approximately 40 mice (20 of each sex) from each congenic and control strain. To eliminate parity and reduce litter size effects only progeny from uniparous dams were characterized and all litters were standardized to 5–7 pups/litter. Mice were weaned at 3 weeks of age. Feed (Purina 5008; 23.5% protein, 6.5% fat, 3.3 Kcal/g) and water were offered *ad libitum*. Mice were weighed to the nearest 0.1 g at 2WK, 3WK, 6WK, and 9WK of age. At 9WK ( ± 5 days) mice were anesthetized under isoflurane and nasal-anal length (NA) and nasal-tail (NT) were measured to the nearest mm. Tail length was calculated as NA minus NT. Anesthetized mice were then sacrificed by decapitation and exsanguinated. Femoral fat pad (FFP), gonadal fat pad (GFP), mesenteric fat pad (MFP) and retroperitoneal fat pad (RFP) were removed and weighed to the nearest mg.

Chemical compositional analysis was performed for HGC, HG11 and HG17 carcasses as previously described with slight modifications [24]. Briefly, after weighing each fat pad was returned to the carcass. The entire gastrointestinal (GI) tract was subsequently removed and carcasses were again weighed. This represented the empty carcass weight (ECW). Carcasses were labeled and secured in two layers of cheesecloth (Fisher) and frozen at -20°C until analysis. At this time, carcasses were freeze-dried for seven days and water content was determined by subtracting the freeze-dried weight from ECW. FAT was then extracted with ether for 7 days, followed by acetone for an additional 7 days in a Soxhlet apparatus. Carcass ash (ASH) was determined measuring the remains after a 16 hour incineration at 575°C. Carcass protein (PROT) was calculated as the remaining portion after carcass fat (FAT) and ASH were subtracted from ECW.

### Statistical analysis

The MEANS and UNIVARIATE procedures of SAS were used to generate descriptive statistics and test normality assumptions for each trait [55]. All data were then analyzed using the GLM procedure of SAS [55] with a linear model that included the fixed effects of strain, sex and strain by sex interaction; dam's weight at breeding by strain was used as a covariate. A second linear model was used to test for strain by *hg *genotype (+/+ or *hg/hg*) interactions. This model included the fixed effects of donor region, sex and HG genotype and all possible two and three-way interactions. Choosing a nominal P value of 0.05 and applying the Bonferroni correction for multiple comparisons established significant differences in the ANOVA's. The critical P values used are indicated in each table.

### Identification of candidate genes

Genes were identified by manual data mining of primary literature, reviews and book chapters. To organize and collate genomic and functional information for each gene we created a custom *Gh *signaling Gene Map Annotator and Pathway Profiler (GenMAPP) pathway [56] (Figure 5). Visualization and color-coding of genes using GenMAPP aided the selection of candidate genes mapping within QTL regions on MMU2, 9, 11 and 17.

### Candidate gene sequencing

PCR amplicons covering the coding sequence and partial 5' and 3' untranslated regions for each selected candidate gene were amplified, purified and sequenced from the CAST strain using protocols outlined in [57] with slight modifications. Total RNA from brain, liver, spleen, lung and testis was isolated from an adult CAST male mouse using Trizol (Ambion). cDNA was produced from total RNA using standard procedures. PCR primer sets for each gene were designed using Primer3 [58] (Additional File 5). The initial amplification was performed in 10 μl PCR reactions using the Invitrogen Platinum TaqPCRx amplification system (Invitrogen). The reactions contained 1X PCR buffer, 1X Enhancer solution, 1.5 mM MgSO_4_, 0.17 mM dNTPs, 1 μM each primer, 0.1 unit of Platinum Taq (Invitrogen) and approximately 25 ng of cDNA. Reactions were incubated for 5 min at 95°C, then cycled for 45 s at 95°C, 45 s at 55°C, 1 min at 72°C for 35 cycles with a final 72°C extension for 10 min on a MJ Research PTC-200. The products were visualized on 1.5% agarose gels containing 0.06 μg/ml EtBr. Bands of the correct size were excised and incubated in 100 μl of sterile H_2_O at 80°C for 10 min to elute DNA. Reamplification reactions consisted of 1X PCR buffer, 1.5 mM MgCl_2_, 0.17 mM dNTPs, 1 μM each primer, 0.1 unit of Taq (Promega) and 10 μl of eluate in a total volume of 50 μl. The same cycling parameters were used for reamplification reactions. Products were visualized on 1% 0.5X TBE agarose gels containing 0.06 μg/ml EtBr, excised and purfied using PCR purification columns (Promega). To quantity each fragment 1 μl of each purified product was run on a 1.0% agarose gel containing 0.06 μg/ml EtBr, along with a DNA mass ladder (Invitrogen). In a 96 well plate, 15 ng of each PCR product was added to 5 pM of primer for sequencing. The College of Agriculture and Environmental Sciences (CAES) Genomics Facility at the University of California, Davis, performed bidirectional sequencing of each amplicon.

### Sequence analysis

B6 mRNA sequences for each gene were downloaded from the May 2004 (mm5) UCSC (NCBI Build 33) genome assembly [28]. These sequences were imported along with all CAST sequence traces into the SeqMan sequence assembly program (DNASTAR) and manually curated for quality. Poor quality reads were resequenced. Individual contigs were created for each gene and polymorphisms were detected and curated by manual inspection.

## Authors' contributions

CRF and JFM conceived the study. CRF developed and characterized each speed congenic strain, assisted with phenotypic data analysis and drafted the manuscript. PMC assisted with experimental design and phenotypic data analysis. JFM assisted in the evaluation of experimental results and the manuscript and provided coordination of the project. All authors read and approved the final manuscript.

## Supplementary Material

Additional File 1 Table of microsatellite markers used in the construction of B6.CAST and HG.CAST speed congenic strains Click here for file

Additional File 2 Table of microsatellite markers used to position the recombinant ends of each speed congenic strain Click here for file

Additional File 3 Table of body weight and growth rate phenotypes of B6.CAST and HG.CAST MMU2 speed congenic strains Click here for file

Additional File 4 Table of results of sequence comparisons between CAST and B6 for MMU2 *hg *modifier candidate genes Click here for file

Additional File 5 Table of PCR primers used to sequence MMU2, 9, 11 and 17 *hg *modifier candidate genes Click here for file

## Figures and Tables

**Table 1:** B6.CAST and HG.CAST speed congenic strains developed to isolate and characterize genome-wide QTL affecting growth and obesity

QTL^a^	MMU	Peak LOD cM (Mbp)^b^	Full congenic name^d^	Abbreviation	Min. interval (Mbp)^e^
*Wg1*	2	31 (52)	B6.CAST-(*D2Mit1-D2Mit160*)N(6)	B62P	3.8–84.8
			HG.CAST-(*D2Mit1-D2Mit160*)N(6)	HG2P	3.8–84.8
			B6.CAST-(*D2Mit322-D2Mit439*)N(6)	B62PM	42.9–92.0
			HG.CAST-(*D2Mit322-D2Mit439*)N(6)	HG2PM	42.9–92.0
***Wg2***	2	59–83^c^	B6.CAST-(*D2Mit329-D2Mit490*)N(6)	B62M	74.9–138.6
*Carp1*		(115–150)	HG.CAST-(*D2Mit329-D2Mit490*)N(6)	HG2M	74.9–138.6
*Cara1*			B6.CAST-(*D2Mit329-D2Mit457*)N(6)	B62D	74.9–181.8
*Feml1*			HG.CAST-(*D2Mit329-D2Mit457*)N(6)	HG2D	74.9–181.8
*Q1Ucd1 -w26*	1	16 (35)	HG.CAST-(*D1Mit118-D1Mit250*)N(6)	HG1	4.0–70.6
*Carfhg1*	5	38 (100)	HG.CAST-(*D5Mit229-D5Mit158*)N(6)	HG5	30.7–114.2
*Wg3*	8	45 (98)	HG.CAST-(*D8Mit9-D8Mit198*)N(6)	HG8	70.2–104.1
***Carfhg2***	9	10 (30)	HG.CAST-(*D9Mit249-D9Mit133*)N(6)	HG9	9.1–84.2
*Feml2*	9	20 (48)	HG.CAST-(*D9Mit249-D9Mit133*)N(6)	HG9	9.1–84.2
***Carp2*** *Wg4* *Cara2*	11	46–50 (80–90)	HG.CAST-(*D11Mit260-D11Mit255*)N(6)	HG11	61.9–114.3
***Feml3*** *Carp3* *Cara3*	17	46–48 (70–76)	HG.CAST-(*D17Mit213-D17Mit142*)N(6)	HG17	14.8–77.3

QTL, quantitative trait locus; MMU, mouse chromosome; LOD, log of the odds

^a ^QTL MGI [59] nomenclature. *Q1Ucd1-w26 *never received an official MGI name, because it only reached a suggestive level of significance; *hg *modifier QTL are in bold [24].

^b ^Mbp position according to the August 2005 mm7 UCSC [28] genome assembly (NCBI Build 35), peak LOD from [24].

^c ^In the linkage analysis performed in [24], the peak location for *Wg2, Carp1, Cara1 *and *Feml1 *was 59–63 cM (115–120 Mbp) in *hg/hg *F_2 _mice and 78–83 cM (135–150 Mbp) in *+/+ *F_2 _mice.

^d ^Full congenic name consists of three parts: 1. B6.CAST, indicates the recipient strain is B6 and the donor strain is CAST; HG.CAST, indicates the recipient strain is HG and the donor strain is CAST; 2. markers defining the minimal congenic interval; 3. the number of backcrosses used for speed congenic development.

^e ^Represents the minimum genomic region spanned by CAST donor alleles.

**Table 2:** Body length and adiposity phenotypes of B6.CAST and HG.CAST MMU2 speed congenic strains

Strain	Sex	N	NA (cm)	Tail (cm)	GFP (g)	FFP (g)	MFP (g)	RFP (g)	TF (g)	AI (%)	BMI
B6C	M	29	9.1 ± 0.04	7.9 ± 0.04	0.344 ± 0.008	0.229 ± 0.006	0.183 ± 0.006	0.066 ± 0.002	0.823 ± 0.018	3.1 ± 0.1	31.6 ± 0.3
B62P	M	20	9.2 ± 0.05	7.9 ± 0.04	**0.309 ± 0.010**	0.235 ± 0.007	0.178 ± 0.007	**0.045 ± 0.003**	0.768 ± 0.023	2.9 ± 0.1	30.8 ± 0.4
B62PM	M	24	9.1 ± 0.05	7.8 ± 0.05	**0.304 ± 0.010**	0.222 ± 0.008	0.173 ± 0.007	**0.046 ± 0.003**	0.746 ± 0.024	2.9 ± 0.1	31.3 ± 0.4
B62M	M	27	8.9 ± 0.07	**7.4 ± 0.06**	**0.294 ± 0.014**	0.239 ± 0.010	**0.214 ± 0.009**	0.066 ± 0.004	0.813 ± 0.032	3.2 ± 0.1	31.4 ± 0.5
B62D	M	21	9.2 ± 0.06	**7.1 ± 0.06**	**0.259 ± 0.013**	**0.177 ± 0.009**	0.154 ± 0.009	**0.038 ± 0.004**	**0.629 ± 0.030**	**2.6 ± 0.1**	**28.9 ± 0.5**

B6C	F	39	8.6 ± 0.03	7.6 ± 0.03	0.166 ± 0.007	0.202 ± 0.005	0.141 ± 0.005	0.027 ± 0.002	0.536 ± 0.016	2.6 ± 0.1	27.7 ± 0.3
B62P	F	23	8.6 ± 0.05	7.8 ± 0.04	0.134 ± 0.010	0.224 ± 0.007	0.148 ± 0.007	0.023 ± 0.003	0.528 ± 0.022	2.7 ± 0.1	**26.1 ± 0.4**
B62PM	F	20	**8.4 ± 0.05**	**7.4 ± 0.05**	0.139 ± 0.011	0.214 ± 0.008	0.154 ± 0.007	0.023 ± 0.003	0.530 ± 0.025	2.8 ± 0.1	26.9 ± 0.4
B62M	F	32	**8.4 ± 0.05**	**7.3 ± 0.05**	0.153 ± 0.010	0.204 ± 0.007	**0.165 ± 0.007**	0.029 ± 0.003	0.551 ± 0.023	2.8 ± 0.1	27.9 ± 0.4
B62D	F	23	8.7 ± 0.06	**7.0 ± 0.06**	**0.110 ± 0.013**	**0.157 ± 0.009**	0.135 ± 0.009	0.019 ± 0.004	**0.420 ± 0.029**	**2.2 ± 0.1**	**25.0 ± 0.5**

HGC	M	31	10.2 ± 0.05	8.0 ± 0.05	0.477 ± 0.013	0.317 ± 0.008	0.297 ± 0.010	0.110 ± 0.005	1.201 ± 0.030	3.3 ± 0.1	35.0 ± 0.4
HG2P	M	18	10.1 ± 0.07	**8.2 ± 0.07**	0.428 ± 0.019	0.326 ± 0.011	**0.227 ± 0.014**	**0.069 ± 0.007**	**1.051 ± 0.043**	3.1 ± 0.1	**32.8 ± 0.5**
HG2PM	M	29	10.1 ± 0.05	8.0 ± 0.05	0.439 ± 0.014	0.303 ± 0.008	**0.244 ± 0.010**	**0.082 ± 0.005**	**1.068 ± 0.031**	3.1 ± 0.1	33.7 ± 0.4
HG2M	M	31	10.1 ± 0.05	**7.4 ± 0.05**	0.469 ± 0.014	0.346 ± 0.008	0.306 ± 0.010	**0.136 ± 0.005**	1.260 ± 0.032	3.6 ± 0.1	33.9 ± 0.4

HGC	F	23	9.6 ± 0.06	7.8 ± 0.06	0.369 ± 0.015	0.305 ± 0.009	0.268 ± 0.011	0.076 ± 0.005	1.020 ± 0.035	3.9 ± 0.1	28.2 ± 0.4
HG2P	F	16	**9.2 ± 0.07**	7.8 ± 0.07	**0.248 ± 0.018**	0.311 ± 0.011	**0.200 ± 0.013**	**0.044 ± 0.006**	**0.804 ± 0.041**	**3.4 ± 0.1**	28.3 ± 0.5
HG2PM	F	36	**9.4 ± 0.04**	7.7 ± 0.04	**0.277 ± 0.012**	**0.265 ± 0.007**	**0.208 ± 0.009**	**0.046 ± 0.004**	**0.797 ± 0.027**	**3.2 ± 0.1**	27.8 ± 0.3
HG2M	F	28	9.6 ± 0.05	**7.2 ± 0.05**	0.329 ± 0.014	**0.269 ± 0.009**	0.247 ± 0.010	0.064 ± 0.005	0.909 ± 0.033	**3.4 ± 0.1**	29.3 ± 0.4

NA, nasal-anal body length; Tail, tail length; GFP, gonadal fat pad; FFP, femoral fat pad; MFP, mesenteric fat pad; RFP, retroperitoneal fat pad; TF, summed weight of four fat pads; AI, adiposity index (TF/body weight*100) and BMI, body mass index (body weight at sacrifice/NA^2 ^*100). Values are expressed as LSMEANS ± SEM. LSMEANS in bold are significantly different than the respective control after Bonferroni correction (B6 (critical P < 0.00625) or HG (P < 0.00833)) within each sex.

**Table 3:** Significance levels for the main effects, two-way and three way interactions of MMU2 speed congenic donor region (DR), HG genotype (HG) (+/+ or *hg/hg*) and sex for selected traits

Donor region (DR)^a^	6WK	9WK	G26	G29	NA	TF	AI
Proximal (2P)							
DR	**<.0001**	**<.0001**	**<.0001**	**<.0001**	**<.0001**	**<.0001**	0.0079
HG	**<.0001**	**<.0001**	**<.0001**	**<.0001**	**<.0001**	**<.0001**	**<.0001**
Sex	**<.0001**	**<.0001**	**<.0001**	**<.0001**	**<.0001**	**<.0001**	0.9840
DR*HG	**<.0001**	0.0094	**<.0001**	0.0181	**<.0001**	**0.0004**	0.0183
DR*sex	0.0287	0.4783	0.0328	0.5938	0.0081	0.8780	0.5218
DR*HG*Sex	0.4092	0.4206	0.6957	0.0882	0.1563	0.1618	0.0219

Proximal middle (2PM)							
DR	**<.0001**	**<.0001**	**<.0001**	**<.0001**	**<.0001**	**<.0001**	**<.0001**
HG	**<.0001**	**<.0001**	**<.0001**	**<.0001**	**<.0001**	**<.0001**	**<.0001**
Sex	**<.0001**	**<.0001**	**<.0001**	**<.0001**	**<.0001**	**<.0001**	0.7248
DR*HG	0.7101	0.4667	0.1277	0.1110	0.7375	**0.0026**	**0.0065**
DR*sex	0.1441	0.3947	0.1095	0.4198	0.0104	0.9323	0.6994
DR*HG*Sex	0.9437	0.2294	0.5748	0.0780	0.7697	**0.0060**	**0.0002**

Middle (2M)							
DR	**0.007**	**<.0001**	0.0234	**0.0002**	**0.0006**	0.1703	0.9707
HG	**<.0001**	**<.0001**	**<.0001**	**<.0001**	**<.0001**	**<.0001**	**<.0001**
Sex	**<.0001**	**<.0001**	**<.0001**	**<.0001**	**<.0001**	**<.0001**	**0.0064**
DR*HG	**0.0057**	0.0563	0.1875	0.6072	0.4329	0.3437	0.0256
DR*sex	0.6968	0.0089	0.8356	0.0207	0.5162	0.1460	0.0437
DR*HG*Sex	0.6205	0.1309	0.6500	0.0656	0.6704	**0.0035**	**0.0004**

6WK, weight at 6 weeks of age; 9WK, weight at 9 weeks of age; G26, weight gain from 2 to 6 weeks of age; G29, weight gain from 2 to 9 weeks of age; NA, nasal-anal body length; TF, total fat pad weight and AI, adiposity index. Significant effects after a Bonferroni correction (critical P < 0.0071) are in bold.

^a ^The distal (2D) donor region was not analyzed.

**Table 4:** Body size, growth rate and length phenotypes of HG.CAST speed congenic strains

Strain	Sex	N	2WK (g)	3WK (g)	6WK (g)	9WK (g)	G26 (g)	G29 (g)	NA (cm)	Tail (cm)
HGC	M	31	7.4 ± 0.2	10.1 ± 0.3	30.4 ± 0.4	36.4 ± 0.4	23.1 ± 0.3	29.0 ± 0.4	10.2 ± 0.05	8.0 ± 0.04
HG1	M	19	6.9 ± 0.3	9.6 ± 0.4	28.8 ± 0.6	34.7 ± 0.6	21.9 ± 0.5	27.8 ± 0.5	**9.9 ± 0.07**	7.8 ± 0.06
HG8	M	15	6.7 ± 0.4	9.2 ± 0.5	29.7 ± 0.8	35.2 ± 0.8	23.1 ± 0.6	28.5 ± 0.7	10.2 ± 0.09	7.9 ± 0.08
HG9	M	29	6.8 ± 0.2	**8.8 ± 0.3**	**27.6 ± 0.5**	34.7 ± 0.5	**20.7 ± 0.4**	27.9 ± 0.4	**9.9 ± 0.05**	**7.8 ± 0.05**
HG11	M	23	7.8 ± 0.3	11.1 ± 0.4	31.7 ± 0.6	38.3 ± 0.6	23.8 ± 0.5	30.4 ± 0.5	10.4 ± 0.07	**8.4 ± 0.06**
HG17	M	10	8.4 ± 0.4	11.4 ± 0.6	29.0 ± 0.9	34.0 ± 1.0	**20.7 ± 0.7**	**25.7 ± 0.8**	9.9 ± 0.11	**7.5 ± 0.09**

HGC	F	23	7.4 ± 0.2	10.1 ± 0.3	23.3 ± 0.5	25.9 ± 0.5	15.9 ± 0.4	18.5 ± 0.4	9.6 ± 0.06	7.8 ± 0.05
HG1	F	31	**6.3 ± 0.2**	**8.2 ± 0.4**	**21.1 ± 0.5**	24.7 ± 0.6	14.8 ± 0.4	18.3 ± 0.5	**9.4 ± 0.06**	**7.5 ± 0.05**
HG8	F	22	6.5 ± 0.4	8.7 ± 0.5	23.1 ± 0.8	26.0 ± 0.8	16.5 ± 0.6	19.5 ± 0.7	9.6 ± 0.09	7.6 ± 0.08
HG9	F	27	7.5 ± 0.2	9.7 ± 0.3	22.6 ± 0.4	25.7 ± 0.5	15.1 ± 0.3	18.2 ± 0.4	**9.3 ± 0.05**	7.6 ± 0.05
HG11	F	24	7.2 ± 0.2	9.6 ± 0.3	22.1 ± 0.5	24.8 ± 0.5	14.8 ± 0.4	17.5 ± 0.5	**9.3 ± 0.06**	8.0 ± 0.05
HG17	F	17	**8.4 ± 0.3**	11.0 ± 0.4	22.0 ± 0.6	25.2 ± 0.7	**13.6 ± 0.5**	16.8 ± 0.5	**9.3 ± 0.07**	**7.4 ± 0.06**

2WK, weight at 2 weeks of age; 3WK, weight at 3 weeks of age; 6WK, weight at 6 weeks of age; 9WK, weight at 9 weeks of age; G26, weight gain from 2 to 6 weeks of age; G29, weight gain from 2 to 9 weeks of age; NA, nasal-anal body length; Tail, tail length. Values are expressed as least square means (LSMEANS) ± SEM. LSMEANS in bold are significantly different than the control (HGC) (critical P < 0.005) after Bonferroni correction within each sex.

**Table 5:** Adiposity phenotypes of HG.CAST speed congenic strains

Strain	Sex	N	GFP (g)	FFP (g)	MFP (g)	RFP (g)	TF (g)	AI (%)	BMI
HGC	M	31	0.477 ± 0.020	0.317 ± 0.011	0.298 ± 0.010	0.110 ± 0.006	1.202 ± 0.042	3.3 ± 0.1	35.0 ± 0.3
HG1	M	19	0.473 ± 0.029	0.353 ± 0.016	0.258 ± 0.014	0.106 ± 0.009	1.190 ± 0.061	3.5 ± 0.2	35.2 ± 0.5
HG8	M	15	0.418 ± 0.038	0.340 ± 0.021	**0.230 ± 0.018**	0.099 ± 0.012	1.087 ± 0.080	3.0 ± 0.2	33.8 ± 0.6
HG9	M	29	**0.615 ± 0.021**	**0.453 ± 0.012**	0.283 ± 0.010	**0.166 ± 0.007**	**1.518 ± 0.045**	**4.3 ± 0.1**	35.6 ± 0.4
HG11	M	23	0.516 ± 0.027	0.312 ± 0.015	0.281 ± 0.013	0.120 ± 0.009	1.229 ± 0.058	3.2 ± 0.2	35.5 ± 0.5
HG17	M	10	0.540 ± 0.043	0.344 ± 0.024	0.278 ± 0.021	0.124 ± 0.014	1.285 ± 0.091	3.8 ± 0.3	35.1 ± 0.8

HGC	F	23	0.370 ± 0.023	0.305 ± 0.013	0.269 ± 0.011	0.076 ± 0.007	1.020 ± 0.048	3.9 ± 0.1	28.1 ± 0.4
HG1	F	31	0.336 ± 0.025	0.328 ± 0.14	**0.205 ± 0.012**	0.055 ± 0.008	0.924 ± 0.053	3.8 ± 0.2	27.9 ± 0.4
HG8	F	22	**0.247 ± 0.037**	0.310 ± 0.021	**0.193 ± 0.018**	0.043 ± 0.012	0.793 ± 0.079	**3.1 ± 0.2**	27.8 ± 0.6
HG9	F	27	**0.624 ± 0.021**	**0.518 ± 0.012**	0.288 ± 0.010	**0.152 ± 0.007**	**1.581 ± 0.045**	**6.1 ± 0.1**	**29.9 ± 0.4**
HG11	F	24	0.436 ± 0.024	0.332 ± 0.014	0.269 ± 0.012	0.087 ± 0.008	1.124 ± 0.052	**4.5 ± 0.2**	28.5 ± 0.4
HG17	F	17	0.410 ± 0.029	0.350 ± 0.016	0.262 ± 0.014	0.086 ± 0.009	1.107 ± 0.062	4.4 ± 0.2	28.9 ± 0.5

GFP, gonadal fat pad; FFP, femoral fat pad; MFP, mesenteric fat pad; RFP, retroperitoneal fat pad; TF, summed weight of four fat pads; AI, adiposity index (TF/body weight*100) and BMI, body mass index (body weight at sacrifice/NA^2 ^*100). Values are expressed as least square means (LSMEANS) ± SEM. LSMEANS in bold are significantly different than the control (HGC) (critical P < 0.005) after Bonferroni correction within each sex.

**Table 6:** Carcass composition phenotypes of HG.CAST speed congenic strains

Line	Sex	N	H_2_O (g)	%H_2_O	FAT (g)	%FAT	ASH (g)	%ASH	PROT (g)	%PROT
HGC	M	31	16.12 ± 0.30	57.74 ± 0.52	2.69 ± 0.11	9.77 ± 0.47	0.86 ± 0.01	3.08 ± 0.03	8.19 ± 0.08	29.41 ± 0.29
HG11	M	22	17.28 ± 0.35	58.62 ± 0.62	2.76 ± 0.13	9.43 ± 0.56	0.85 ± 0.01	**2.89 ± 0.03**	8.51 ± 0.10	29.06 ± 0.30
HG17	M	10	**14.27 ± 0.55**	55.32 ± 0.97	2.87 ± 0.21	11.34 ± 0.88	**0.78 ± 0.02**	3.02 ± 0.05	7.80 ± 0.15	30.33 ± 0.35

HGC	F	23	10.08 ± 0.32	51.76 ± 0.56	2.66 ± 0.12	13.83 ± 0.51	0.68 ± 0.01	3.50 ± 0.03	5.95 ± 0.09	30.91 ± 0.27
HG11	F	22	8.95 ± 0.33	**48.96 ± 0.58**	2.94 ± 0.12	**16.02 ± 0.53**	**0.58 ± 0.01**	**3.20 ± 0.03**	5.80 ± 0.09	31.82 ± 0.32
HG17	F	17	8.88 ± 0.38	**48.91 ± 0.67**	2.84 ± 0.14	15.69 ± 0.61	**0.60 ± 0.02**	**3.31 ± 0.04**	5.80 ± 0.11	**32.09 ± 0.50**

H_2_O, carcass water; %H_2_O, water as a percent of carcass weight; FAT, carcass fat; %FAT, fat as a percent of carcass weight; ASH, carcass ash; %ASH, ash as a percent of carcass weight; PROT, carcass protein, %PROT, protein as a percent of carcass weight. Values are expressed as least square means (LSMEANS) ± SEM. LSMEANS in bold are significantly different than the control (HGC) (critical P < 0.0125) after Bonferroni correction within each sex.

**Table 7:** Nonsynonymous SNP in MMU2, 9 and 17 *hg *modifier candidate genes predicted by SIFT and Polyphen to alter protein function

Gene	MMU	Mbp	AAÄ^a^	B6/CAST^b ^Polyphen	CAST/B6 Polyphen	B6/CAST SIFT	CAST/B6 SIFT
*Nmi*	2	52.0	R243C	B	B	T	D
*Mtx2*	2	74.7	T1099M	PSD	PSD	T	D
*Ubr1*	2	120.6	A1371T	B	B	D	T
*Ptpns1*	2	129.3	H224N	PBD	PBD	T	T
*Ubce7ip5*	2	130.3	A314P	PSD	PSD	T	T
	2		I366T	B	B	T	D
*Plcg1*	2	160.5	T1165A	B	B	T	D
*Mmp9*	2	164.7	A514P	B	B	D	D
	2		P639L	PBD	PBD	T	T
*Tyk2*	9	21.0	R234H	PSD	PSD	T	T
			C831S	PSD	PSD	T	T
			T1099M	PSD	PSD	T	D
*Vav*	17	55.5	Q29H	PSD	PBD	T	T
			Q53H	PBD	PSD	T	T
			Q65H	PBD	PSD	T	D

MMU, mouse chromosome; AA, amino acid; PBD, probably damaging, PSD, possibly damaging; B, Benign; T, tolerated and D, deleterious.

^a ^Amino acid change, first residue listed corresponds to the CAST allele and second corresponds to the B6 allele.

^b ^Indicates which allele was designated as "mutant", i.e. B6/CAST, B6 allele is mutant and CAST is wild type.
