# Supplementary material for: Genome-wide isolation of growth and obesity QTL using mouse speed congenic strains
Source: BMC Genomics. 2006 May 2;7:102. doi: 10.1186/1471-2164-7-102 (PMC1482699; doi:10.1186/1471-2164-7-102)
Supplement: Additional File 1 — Table of microsatellite markers used in the construction of B6.CAST and HG.CAST speed congenic strains [file 1471-2164-7-102-S1.doc]

**Additional Table 1.** Microsatellite markers used in the construction of B6.CAST and HG.CAST speed congenic strains

| Marker ^a^ | MMU | cM^b^ | Mbp^c^ | CAST | B6/HG | PCR | Marker | MMU | cM^b^ | Mbp^c^ | CAST | B6/HG | PCR |
| --- | --- | --- | --- | --- | --- | --- | --- | --- | --- | --- | --- | --- | --- |
| *D10Mit44* | 1 |  | 16.3 | 172 | 122 | 55/3.0 | *D9Mit60* | 9 | 4.4 | 15.3 | 156 | 210 | 58/1.5 |
| *D1Mit4* | 1 | 8.7 | 21.3 | 168 | 200 | 55/1.5 | *D9Mit188* | 9 | 7.7 | 31.3 | 160 | 134 | 55/1.5 |
| *D12Mit63* | 1 |  | 22.9 | 200 | 160 | 58/1.5 | *D9Mit94* | 9 | 23 | 49.5 | 260 | 300 | 55/1.5 |
| *D1Mit432* | 1 | 12 | 24.5 | 136 | 96 | 55/1.5 | *D9Mit262* | 9 | 39.3 | 75.5 | 102 | 142 | 55/2.0 |
| *D1Mit172* | 1 | 20.8 | 41.0 | 165 | 143 | 55/3.0 | *D9Mit278* | 9 | 61.2 | 107.4 | 172 | 124 | 55/1.5 |
| *D1Mit480* | 1 | 32.8 | 64.1 | 137 | 170 | 55/1.5 | *D10Mit259* | 10 | 33.9 | 72.3 | 153 | 113 | 55/1.5 |
| *D1Mit415* | 1 | 51.4 | 86.3 | 111 | 157 | 55/1.5 | *D10Mit270* | 10 | 75.4 | 125.5 | 146 | 108 | 58/1.5 |
| *D1Mit311* | 1 | 66.7 | 123.3 | 208 | 156 | 55/1.5 | *D11Mit230* | 11 | 13.1 | 28.0 | 184 | 224 | 58/1.5 |
| *D1Mit111* | 1 | 91.8 | 169.0 | 202 | 170 | 55/3.0 | *D11Mit5* | 11 | 36.1 | 67.3 | 142 | 219 | 55/1.5 |
| *D2Mit362* | 2 | 10.9 | 19.0 | 147 | 115 | 55/3.0 | *D11Mit36* | 11 | 43.7 | 83.9 | 302 | 234 | 55/1.5 |
| *D2Mit72* | 2 | 29.5 | 49.3 | 188 | 144 | 55/3.0 | *D11Mit67* | 11 | 60.1 | 97.1 | 178 | 134 | 58/1.5 |
| *D2Mit205* | 2 | 38.3 | 65.2 | 127 | 85 | 55/1.5 | *D11Mit255* | 11 | 75.4 | 114.3 | 208 | 188 | 55/3.0 |
| *D2Mit93* | 2 | 44.8 | 76.7 | 178 | 142 | 55/1.5 | *D12Mit4* | 12 | 28.4 | 77.0 | 269 | 202 | 55/1.5 |
| *D2Mit17* | 2 | 56.8 | 122.5 | 242 | 205 | 55/1.5 | *D12Mit101* | 12 | 44.8 | 100.0 | 126 | 170 | 55/1.5 |
| *D2Mit260* | 2 | 68.9 | 148.9 | 140 | 170 | 55/1.5 | *D13Mit84* | 13 | 4.4 | 25.0 | 272 | 220 | 55/1.5 |
| *D2Mit456* | 2 | 86.3 | 168.7 | 200 | 130 | 55/1.5 | *D13Mit253* | 13 | 24 | 61.8 | 117 | 78 | 55/3.0 |
| *D2Mit148* | 2 | 91.8 | 179.3 | 217 | 117 | 55/1.5 | *D13Mit290* | 13 | 40.4 | 100.0 | 106 | 152 | 50/1.5 |
| *D3Mit240* | 3 | 13.1 | 32.4 | 150 | 138 | 55/1.5 | *D14Mit206* | 14 | 2.2 | 18.4 | 166 | 126 | 55/2.0 |
| *D3Mit189* | 3 | 37.2 | 100.8 | 182 | 138 | 55/1.5 | *D14Mit45* | 14 | 19.7 | 31.8 | 140 | 106 | 55/1.5 |
| *D3Mit200* | 3 | 55.7 | 147.9 | 99 | 131 | 55/1.5 | *D14Mit37* | 14 | 36.1 | 57.3 | 72 | 136 | 58/1.5 |
| *D3Mit219* | 3 | 64.5 | 154.9 | 203 | 149 | 55/1.5 | *D14Mit71* | 14 | 52.5 | 90.6 | 132 | 172 | 55/1.5 |
| *D4Mit236* | 4 | 16.4 | 39.3 | 160 | 204 | 55/1.5 | *D15Mit177* | 15 | 6.6 | 12.5 | 188 | 120 | 55/1.5 |
| *D4Mit15* | 4 | 39.3 | 90.9 | 313 | 279 | 55/1.5 | *D15Mit230* | 15 | 21.9 | 54.9 | 162 | 126 | 55/3.0 |
| *D4Mit203* | 4 | 60.1 | 129.0 | 89 | 144 | 55/1.5 | *D15Mit71* | 15 | 35 | 79.7 | 150 | 118 | 55/3.0 |
| *D5Mit353* | 5 | 16.4 | 39.7 | 156 | 122 | 55/1.5 | *D15Mit223* | 15 | 55.7 | 93.0 | 253 | 325 | 55/2.0 |
| *D5Mit111* | 5 | 27.3 | 67.2 | 171 | 145 | 55/1.5 | *D16Mit29* | 16 | 15.3 | 19.8 | 172 | 148 | 55/2.0 |
| *D5Mit311* | 5 | 32.8 | 89.0 | 80 | 120 | 55/2.0 | *D16Mit61* | 16 | 29.5 | 48.1 | 201 | 151 | 55/1.5 |
| *D5Mit51* | 5 | 74.3 | 141.1 | 166 | 142 | 55/1.5 | *D16Mit49* | 16 | 39.3 | 78.2 | 172 | 146 | 55/2.0 |
| *D6Mit354* | 6 | 13.1 | 53.3 | 126 | 172 | 55/1.5 | *D17Mit28* | 17 | 7.7 | 32.1 | 86 | 120 | 55/3.0 |
| *D6Mit29* | 6 | 27.3 | 86.9 | 212 | 124 | 55/1.5 | *D17Mit203* | 17 | 29.5 | 57.9 | 158 | 122 | 55/1.5 |
| *D6Mit287* | 6 | 44.8 | 112.2 | 162 | 88 | 50/1.5 | *D17Mit142* | 17 | 41.5 | 77.3 | 111 | 147 | 50/1.5 |
| *D7Mit112* | 7 | 5.5 | 23.4 | 176 | 144 | 55/1.5 | *D18Mit202* | 18 | 14.2 | 43.8 | 143 | 111 | 55/1.5 |
| *D7Mit200* | 7 | 27.3 | 76.0 | 183 | 151 | 55/1.5 | *D18Mit4* | 18 | 37.2 | 84.5 | 180 | 210 | 55/1.5 |
| *D7Mit164* | 7 | 45.9 | 119.5 | 275 | 309 | 55/3.0 | *D19Mit69* | 19 | 8.7 | 13.6 | 181 | 137 | 50/3.0 |
| *D8Mit356* | 8 | 8.7 | 25.3 | 509 | 247 | 55/1.5 | *D19Mit19* | 19 | 26.2 | 39.3 | 98 | 142 | 55/1.5 |
| *D8Mit339* | 8 | 25.1 | 39.1 | 84 | 122 | 50/1.5 | *D19Mit34* | 19 | 44.8 | 55.0 | 200 | 156 | 55/2.0 |
| *D8Mit234* | 8 | 38.3 | 80.7 | 96 | 126 | 55/2.0 | *DXMit54* | X | 2.2 | 10.3 | 154 | 192 | 50/1.5 |
| *D8Mit32* | 8 | 44.8 | 93.5 | 186 | 134 | 55/1.5 | *DXMit18* | X | 29.5 | 99.0 | 190 | 148 | 55/1.5 |
| *D8Mit211* | 8 | 50.3 | 101.3 | 186 | 154 | 55/1.5 | *DXMit197* | X | 50.3 | 146.3 | 160 | 122 | 55/1.5 |
| *D8Mit200* | 8 | 60.1 | 113.3 | 233 | 197 | 55/2.0 |  |  |  |  |  |  |  |

MMU, mouse chromosome; cM, centimorgan; Mbp, megabase pairs; CAST, microsatellite allele size in CAST/EiJ; B6/HG, microsatellite allele size in B6 and HG; PCR, PCR conditions: annealing temperature/[MgCl_2_].

^a^ Markers *D10Mit44 and D12Mit63* have been localized to MMU10 and 12, respectively, on the MIT F_2_ mouse genetic map. However, during construction of the B6.CAST1 speed congenic strain these markers were always heterozygous after each backcross, suggesting they were located on MMU1. Subsequent BLAT analysis of the microsatellite flanking sequence using the mouse genome assembly confirmed both markers are located on MMU1.

^b^ cM position based on MIT F_2_ intercross data. Data obtained from Broad Institute, MIT: Genetic and Physical Maps of the Mouse Genome, http://www.broad.mit.edu/cgi-bin/mouse/index.

^c^ Mbp position from the August 2005 UCSC [28] mm7 genome assembly (NCBI Build 35).
