# Supplementary material for: Genome-wide isolation of growth and obesity QTL using mouse speed congenic strains
Source: BMC Genomics. 2006 May 2;7:102. doi: 10.1186/1471-2164-7-102 (PMC1482699; doi:10.1186/1471-2164-7-102)
Supplement: Additional File 2 — Table of microsatellite markers used to position the recombinant ends of each speed congenic strain [file 1471-2164-7-102-S2.doc]

**Additional Table 2.** Microsatellite markers used to position the recombinant ends of each speed congenic strain

| Congenic | Marker | cM^a^ | Mbp^b^ | CAST | B6/HG | PCR | Genotype^c^ |
| --- | --- | --- | --- | --- | --- | --- | --- |
| B62P/HG2P | *D2Mit1* | 2.2 | 3.8 | 140 | 124 | 55/1.5 | CAST |
|  | *D2Mit4* | 7.7 | 10.0 | 166 | 190 | 55/1.5 | CAST |
|  | *D2Mit183* | 44.8 | 77.4 | 129 | 145 | 55/1.5 | CAST |
|  | *D2Mit94* | 48.1 | 80.0 | 188 | 160 | 55/1.5 | CAST |
|  | *D2Mit160* | 48.1 | 84.8 | 147 | 131 | 55/1.5 | CAST |
|  | *D2Mit45* | 50.3 | 91.8 | 146 | 120 | 55/1.5 | B6/HG |
| B62PM/HG2PM | *D2Mit466* | 27.3 | 41.2 | 160 | 114 | 55/1.5 | B6/HG |
|  | *D2Mit322* | 28.4 | 42.9 | 112 | 128 | 55/1.5 | CAST |
|  | *D2Mit88* | 29.5 | 45.5 | 166 | 184 | 55/1.5 | CAST |
|  | *D2Mit439* | 50.3 | 92.0 | 92 | 118 | 55/2.0 | CAST |
|  | *D2Mit130* | 50.3 | 97.4 | 236 | 218 | 55/2.0 | B6/HG |
| B62M/HG2M | *D2Mit37* | 44.8 | 74.5 | 196 | 174 | 55/1.5 | B6/HG |
|  | *D2Mit329* | 44.8 | 74.9 | 144 | 126 | 55/1.5 | CAST |
|  | *D2Mit399* | 60.1 | 128.8 | 108 | 91 | 55/2.0 | CAST |
|  | *D2Mit490* | 64.5 | 138.6 | 108 | 88 | 55/2.0 | CAST |
|  | *D2Mit166* | 65.6 | 140.8 | 157 | 119 | 55/2.0 | B6/HG |
|  | *D2Mit212* | 65.6 | 141.7 | 173 | 151 | 55/1.5 | B6/HG |
| B62D/HG2D | *D2Mit37* | 44.8 | 74.5 | 196 | 174 | 55/1.5 | B6/HG |
|  | *D2Mit329* | 44.8 | 74.9 | 144 | 126 | 55/1.5 | CAST |
|  | *D2Mit457* | 98.4 | 181.8 | 140 | 120 | 55/1.5 | CAST |
| HG1 | *D1Mit118* | 4.4 | 4.0 | 223 | 205 | 55/2.0 | CAST |
|  | *D1Mit250* | 37.2 | 70.6 | 117 | 150 | 55/2.0 | CAST |
| HG5 | *D5Mit229* | 14.2 | 30.7 | 100 | 150 | 55/2.0 | CAST |
|  | *D5Mit89* | 36.1 | 99.5 | 180 | 148 | 55/2.0 | CAST |
|  | *D5Mit240* | 43.7 | 108.3 | 178 | 156 | 55/2.0 | CAST |
|  | *D5Mit158* | 48.1 | 114.2 | 321 | 400 | 55/1.5 | CAST |
|  | *D5Mit29* | 62.3 | 130.1 | 192 | 158 | 55/1.5 | HG |
| HG8 | *D8Mit9* | 35.0 | 70.2 | 118 | 151 | 55/2.0 | CAST |
|  | *D8Mit198* | 52.5 | 104.1 | 162 | 202 | 55/2.0 | CAST |
| HG9 | *D9Mit249* | 2.2 | 9.1 | 144 | 128 | 55/2.0 | CAST |
|  | *D9Mit208* | 33.9 | 62.3 | 82 | 112 | 55/1.5 | CAST |
|  | *D9Mit133* | 41.5 | 84.2 | 159 | 131 | 55/2.0 | CAST |
|  | *D9Mit10* | 43.7 | 89.6 | 178 | 75 | 55/2.0 | HG |
|  | *D9Mit36* | 53.6 | 100.1 | 296 | 220 | 55/2.0 | HG |
| HG11 | *D11Mit313* | 25.1 | 56.2 | 150 | 124 | 55/2.0 | HG |
|  | *D11Mit260* | 31.7 | 61.9 | 118 | 98 | 55/2.0 | CAST |
|  | *D11Mit293* | 78.7 | 120.3 | 100 | 114 | 55/2.0 | HG |
| HG17 | *D17Mit213* | 3.3 | 14.8 | 142 | 124 | 55/2.0 | CAST |
|  | *D17Mit123* | 50.3 | 91.6 | 165 | 133 | 55/2.0 | HG |

Abbreviations: cM, centimorgan; Mbp, megabase pairs; CAST, microsatellite allele size in CAST/EiJ; B6/HG, microsatellite allele size in B6 and HG; PCR, PCR conditions: annealing temperature/[MgCl_2_]

^a^ cM position based on the MIT F_2_ intercross, data obtained from [[http://www.broad.mit.edu/cgi-bin/mouse /index#genetic](http://www.broad.mit.edu/cgi-bin/mouse /index" \l "genetic)].

^b^ Mbp from the August 2005 UCSC [28] mm7 genome assembly (NCBI Build 35).

^c^ Congenic genotype of each marker.
