# Supplementary material for: Genome-wide isolation of growth and obesity QTL using mouse speed congenic strains
Source: BMC Genomics. 2006 May 2;7:102. doi: 10.1186/1471-2164-7-102 (PMC1482699; doi:10.1186/1471-2164-7-102)
Supplement: Additional File 3 — Table of body weight and growth rate phenotypes of B6.CAST and HG.CAST MMU2 speed congenic strains [file 1471-2164-7-102-S3.doc]

**Additional Table 3.** Body weight and growth rate phenotypes of B6.CAST and HG.CAST MMU2 speed congenic strains

| Strain | Sex | N | 2WK (g) | 3WK (g) | 6WK (g) | 9WK (g) | G26 (g) | G29 (g) |
| --- | --- | --- | --- | --- | --- | --- | --- | --- |
| B6C | M | 29 | 7.4±0.1 | 10.3±0.2 | 22.9±0.2 | 26.1±0.2 | 15.5±0.2 | 18.8±0.2 |
| B62P | M | 20 | 7.2±0.1 | 10.2±0.2 | 23.0±0.3 | 25.9±0.3 | 15.8±0.2 | 18.7±0.3 |
| B62PM | M | 24 | 6.9±0.1 | 9.7±0.2 | 22.1±0.3 | 25.7±0.3 | 15.2±0.2 | 18.8±0.3 |
| B62M | M | 27 | 7.0±0.2 | 10.1±0.3 | 21.8±0.4 | **24.8±0.4** | 14.8±0.3 | 17.8±0.4 |
| B62D | M | 21 | **5.8±0.2** | **7.6±0.3** | **20.5±0.3** | **24.1±0.4** | 14.8±0.3 | 18.4±0.3 |
| B6C | F | 39 | 7.1±0.1 | 9.6±0.2 | 18.5±0.2 | 20.4±0.2 | 11.5±0.1 | 13.3±0.2 |
| B62P | F | 23 | 7.1±0.1 | 9.9±0.2 | 17.9±0.3 | **19.2±0.3** | **10.7±0.2** | **12.1±0.3** |
| B62PM | F | 20 | 6.7±0.2 | 9.0±0.2 | **17.1±0.3** | **18.9±0.3** | **10.4±0.2** | **12.2±0.3** |
| B62M | F | 32 | 6.9±0.1 | 9.5±0.2 | 17.7±0.3 | 19.5±0.3 | 10.9±0.2 | 12.7±0.3 |
| B62D | F | 23 | **5.6±0.2** | **7.3±0.3** | **17.0±0.3** | **18.7±0.4** | 11.4±0.3 | 13.0±0.3 |
| HGC | M | 31 | 7.4±0.2 | 10.1±0.2 | 30.4±0.3 | 36.4±0.5 | 23.1±0.3 | 29.0±0.4 |
| HG2P | M | 18 | 7.3±0.2 | 9.9±0.3 | **28.6±0.5** | **33.8±0.6** | **21.2±0.4** | **26.5±0.6** |
| HG2PM | M | 29 | 7.4±0.2 | 10.0±0.2 | **29.1±0.4** | **34.1±0.5** | **21.7±0.3** | **26.8±0.5** |
| HG2M | M | 31 | 7.5±0.2 | 10.4±0.2 | 30.4±0.4 | **34.5±0.5** | 22.9±0.3 | **27.0±0.5** |
| HGC | F | 23 | 7.4±0.2 | 10.1±0.3 | 23.3±0.4 | 25.9±0.5 | 15.9±0.4 | 18.5±0.5 |
| HG2P | F | 16 | 6.8±0.2 | 9.2±0.3 | **20.1±0.5** | **23.7±0.6** | **13.4±0.4** | 16.9±0.6 |
| HG2PM | F | 36 | 7.2±0.2 | 9.7±0.2 | **21.7±0.3** | 24.4±0.4 | **14.5±0.3** | 17.2±0.4 |
| HG2M | F | 28 | 7.7±0.2 | 10.2±0.2 | 23.6±0.4 | 26.6±0.5 | 15.8±0.3 | 18.9±0.5 |

2WK, weight at 2 weeks of age; 3WK, weight at 3 weeks of age; 6WK, weight at 6 weeks of age; 9WK, weight at 9 weeks of age; G26, weight gain from 2 to 6 weeks of age; G29, weight gain from 2 to 9 weeks of age; Values are expressed as LSMEANS ± SEM. LSMEANS in bold are significantly different than the respective control after Bonferroni correction (B6 (critical P<0.00625) or HG (P<0.00833)) within each sex.
