# Supplementary material for: Genome-wide isolation of growth and obesity QTL using mouse speed congenic strains
Source: BMC Genomics. 2006 May 2;7:102. doi: 10.1186/1471-2164-7-102 (PMC1482699; doi:10.1186/1471-2164-7-102)
Supplement: Additional File 4 — Table of results of sequence comparisons between CAST and B6 for MMU2 hg modifier candidate genes [file 1471-2164-7-102-S4.doc]

**Additional Table 4.** Results of sequence comparisons between CAST and B6 for MMU2 *hg* modifier candidate genes

| Gene Symbol | Gene name | MMU | Mbp^a^ | Accession  number | Length^b^ | CDS^c^ | %CDS^d^ | AA^e^ | Polymorphism | | |
| --- | --- | --- | --- | --- | --- | --- | --- | --- | --- | --- | --- |
|  |  |  |  |  |  |  |  |  | SNP | INDEL^f^ | AA |
| *Nmi* | N-myc (and STAT) interactor | 2 | 52.0 | [GenBank:AY902321] | 983 | 945 | 100 | 314 | 9 |  | 4 |
| *Stam2* | signal transducing adaptor molecule 2 | 2 | 52.7 | [GenBank:AY902333] | 2034 | 1572 | 100 | 523 | 6 |  | 0 |
| *Sp3* | trans-acting transcription factor 3 | 2 | 72.8 | [GenBank:AY902330] | 2205 | 2068 | 95 | 688 | 3 |  | 0 |
| *Atf2* | activating transcription factor 2 | 2 | 73.7 | [GenBank:AY902311] | 1398 | 1344 | 100 | 447 | 0 |  | 0 |
| *Mtx2* | Metaxin 2 | 2 | 74.7 | [GenBank:AY902354] | 1391 | 792 | 100 | 263 | 2 |  | 1 |
| *Dusp19* | dual specificity phosphatase 19 | 2 | 80.5 | [GenBank:AY902341] | 960 | 663 | 100 | 220 | 0 |  | 0 |
| *Psmc3* | proteasome (prosome, macropain) 26S subunit | 2 | 90.9 | [GenBank:AY902337] | 1426 | 1329 | 100 | 442 | 3 |  | 0 |
| *Sgne1* | secretory granule neuroendocrine protein 1 (7B2) | 2 | 113.6 | [GenBank:AY902313] | 1006 | 639 | 100 | 212 | 7 |  | 0 |
| *Plcb2* | phospholipase C beta 2 | 2 | 118.5 | [GenBank:AY902324] | 2256 | 2109 | 100 | 702 | 3 |  | 0 |
| *Ubr1* | ubiquitin protein ligase E3 component n-recognin 1 | 2 | 120.6 | [GenBank:AY902334] | 5849 | 5274 | 100 | 1757 | 25 | 1 I | 8 |
| *Dusp2* | dual specificity phosphatase 2 | 2 | 127.1 | [GenBank:AY902315] | 1064 | 957 | 100 | 318 | 2 |  | 1 |
| *Il1a* | interleukin 1 alpha | 2 | 129.0 | [GenBank:AY902318] | 999 | 813 | 100 | 270 | 0 |  | 0 |
| *Il1b* | interleukin 1 beta | 2 | 129.1 | [GenBank:AY902319] | 1034 | 810 | 100 | 269 | 4 | 1 I | 0 |
| *Ptpns1* | protein tyrosine phosphatase, non-receptor type | 2 | 129.3 | [GenBank:AY902327] | 2023 | 1530 | 100 | 509 | 25 |  | 17 |
| *Ptpra* | protein tyrosine phosphatase, receptor type, A | 2 | 130.2 | [GenBank:AY902328] | 2402 | 2382 | 100 | 793 | 6 |  | 0 |
| *Ubce7ip5* | ubiquitin conjugating enzyme 7 interacting protein 5 | 2 | 130.3 | [GenBank:AY902351] | 2483 | 1620 | 100 | 539 | 14 |  | 6 |
| *Plcb1* | phospholipase C beta 1 | 2 | 134.5 | [GenBank:AY902323] | 3573 | 3473 | 95 | 1158 | 17 |  | 0 |
| *Plcb4* | phospholipase C beta 4 | 2 | 135.5 | [GenBank:AY902325] | 3608 | 3528 | 100 | 1175 | 13 |  | 0 |
| *Pcsk2* | proprotein convertase subtilisin/kexin type 2 | 2 | 143.3 | [GenBank:AY902322] | 2052 | 1914 | 100 | 637 | 11 |  | 0 |
| *Foxa2* | forkhead box A2 | 2 | 147.8 | [GenBank:AY902316] | 1951 | 1380 | 100 | 459 | 3 |  | 1 |
| *Sstr4* | somatostatin receptor 4 | 2 | 148.1 | [GenBank:AY902332] | 1565 | 1158 | 100 | 385 | 2 |  | 0 |
| *Bcl2l1* | Bcl2-like 1 | 2 | 152.5 | [GenBank:AY902314] | 1448 | 702 | 100 | 233 | 2 |  | 0 |
| *Scand1* | SCAN domain-containing 1 | 2 | 156.0 | [GenBank:AY902329] | 469 | 429 | 100 | 142 | 0 |  | 0 |
| *Ghrh* | growth hormone releasing hormone | 2 | 157.0 | [GenBank:AY902312] | 449 | 312 | 100 | 103 | 3 |  | 0 |
| *Src* | rous sarcoma oncogene | 2 | 157.2 | [GenBank:AY902331] | 2355 | 1626 | 100 | 541 | 2 |  | 0 |
| *Plcg1* | phospholipase C gamma 1 | 2 | 160.5 | [GenBank:AY902326] | 3658 | 3537 | 100 | 1178 | 6 |  | 1 |
| *Hnf4a* | hepatic nuclear factor 4 alpha | 2 | 163.3 | [GenBank:AY902317] | 1866 | 1425 | 100 | 474 | 12 | 2 I | 0 |
| *Mmp9* | matrix metalloproteinase 9 | 2 | 164.7 | [GenBank:AY902320] | 2765 | 2193 | 100 | 730 | 14 | 1 I | 4 |
| *Tyk2* | tyrosine kinase 2 | 9 | 21.0 | [GenBank:AY902350] | 3753 | 3555 | 100 | 1184 | 16 |  | 5 |
| *Cbl* | Casitas B-lineage lymphoma | 9 | 44.1 | [GenBank:AY902338] | 2846 | 2742 | 100 | 913 | 8 |  | 1 |
| *Cul5* | cullin 5 | 9 | 53.6 | [GenBank:AY902340] | 2766 | 2343 | 100 | 780 | 5 | 1 I | 0 |
| *Ptpn9* | protein tyrosine phosphatase, non-receptor type | 9 | 57.0 | [GenBank:AY902347] | 3047 | 1782 | 100 | 593 | 6 | 1 I | 0 |
| *Csk* | c-src tyrosine kinase (Csk) | 9 | 57.7 | [GenBank:AY902339] | 2062 | 1353 | 100 | 450 | 7 |  | 0 |
| *Pias1* | *protein inhibitor of activated STAT 1* | 9 | 62.9 | [GenBank:AY902346] | 2018 | 1956 | 100 | 651 | 0 |  | 0 |
| *Map2k1* | mitogen activated protein kinase kinase 1 | 9 | 64.2 | [GenBank:AY902345] | 2028 | 1182 | 100 | 393 | 2 |  | 0 |
| *Onecut1* | one cut domain, family member 1 | 9 | 74.9 | [GenBank:AY902344] | 1397 | 1326 | 100 | 441 | 0 |  | 0 |
| *Slc2a4* | Solute carrier family 2, facilitated glucose transporter, member 4 | 11 | 70.0 | [GenBank:AY902342] | 2009 | 1530 | 100 | 509 | 4 |  | 0 |
| *Crk* | v-crk sarcoma virus CT10 oncogene homolog | 11 | 75.8 | [GenBank:AY902336] | 2252 | 915 | 100 | 304 | 2 | 1 I | 0 |
| *Ccl9* | chemokine (C-C motif) ligand 9 | 11 | 83.6 | [GenBank:AY902335] | 995 | 369 | 100 | 122 | 8 |  | 1 |
| *Tcf2* | transcription factor 2 | 11 | 83.9 | [GenBank:AY902343] | 2051 | 1677 | 100 | 558 | 5 | 3 D | 1 |
| *Vav* | vav 1 oncogene | 17 | 55.5 | [GenBank:AY902352] | 2812 | 2538 | 100 | 845 | 15 |  | 5 |
| *Sos1* | Son of sevenless protein homolog 1 | 17 | 78.7 | [GenBank:AY902349] | 5060 | 3525 | 100 | 1174 | 11 | 1 I | 0 |
| *Socs5* | suppressor of cytokine signaling 5 | 17 | 85.4 | [GenBank:AY902348] | 3274 | 1611 | 100 | 536 | 12 | 1 I | 0 |
| *Calm2* | calmodulin 2 | 17 | 85.7 | [GenBank:AY902353] | 850 | 450 | 100 | 149 | 0 |  | 0 |

MMU, mouse chromosome; CDS, coding sequence; AA, amino acid; SNP, single nucleotide polymorphism; INDEL, insertion/deletion polymorphism.

^a^ Mbp position from the August 2005 mm7 UCSC [28] genome assembly (NCBI Build 35).

^b^ Number of bp sequenced for each gene.

^c^ Length of CDS for each gene.

^d^ Percent of CDS sequenced for each gene.

^e^ Number of amino acids in the protein product of each gene.

^f^ I indicates an insertion of one or more base pairs, all insertions were in UTR sequence.
