# Supplementary material for: Genome-wide isolation of growth and obesity QTL using mouse speed congenic strains
Source: BMC Genomics. 2006 May 2;7:102. doi: 10.1186/1471-2164-7-102 (PMC1482699; doi:10.1186/1471-2164-7-102)
Supplement: Additional File 5 — Table of PCR primers used to sequence MMU2, 9, 11 and 17 hg modifier candidate genes [file 1471-2164-7-102-S5.doc]

**Additional Table 5.** PCR primers used to sequence MMU2, 9, 11 and 17 *hg* modifier candidate genes

| Name | Sequence | Name | Sequence |
| --- | --- | --- | --- |
| Nmi-A-F | ATGGAGCAGAAGCATCCTGT | Nmi-A-R | TCAGACAGCTCATCGGGAAT |
| Nmi-B-F | TCATGTCGTGCAGATGGAAG | Nmi-B-R | TGGCAGTGATTTTAGTTCAAGG |
| Stam2-A-F | CAGAGCGTCGGGATTCAG | Stam2-A-R | TCCGTGTACTGCTGCTTCTG |
| Stam2-B-F | CGGGACATCATTGAACAAAA | Stam2-B-R | GCTTCCAGCACCTTTACGTT |
| Stam2-C-F | CAACAGATGGGTCCGATGAT | Stam2-C-R | AGGTCCTGGTGCGTCTCTAC |
| Stam2-D-F | AGAACACAGCATCCGGTTTG | Stam2-D-R | GGAGCCAACAGGAAATATGC |
| Sp3-A-F | CTTCGCTTCTCACCATGTGTA | Sp3-A-R | CCAGAGGCGAGTAAGGTTTG |
| Sp3-B-F | ACGCTCAGCAGGTTCAGATT | Sp3-B-R | CAGAAACAGGCGACTGGATA |
| Sp3-C-F | TGCCAACATCCTCTTCATCA | Sp3-C-R | TGGCAACTGACCAGTGCTTA |
| Sp3-D-F | TCAGACACTCACGCTTGGTC | Sp3-D-R | GCGTTTTGAACATTCTGGAC |
| Sp3-E-F | GGAGCGCCCTTTTATTTGTA | Sp3-E-R | GGCATTTCAGTGTCATGTATAACC |
| ATF-P1 | GGACCTGTGGAATATGAGTGATGAC | ATF-P2 | TCAACTGCTGCTATACCAACTCAG |
| Mtx2-A-F | aggtgagctctgctcgtcttag | Mtx2-A-R | TCACTTCCCACTGCTTCTGATA |
| Mtx2-B-F | CTCCAGTGGTGTGATGAAGC | Mtx2-B-R | ttggttttgagatcctgcaac |
| Mtx2-C-F | AGACTGGGGTAAAGGCAGGT | Mtx2-C-R | cgctggttaacgcagtttag |
| Dusp19-A-F | GGAAGACACGCCCAGATAAA | Dusp19-A-R | CAGGGCTTAATAACGCCAAC |
| Dusp19-B-F | GGGAAGATGCCACAGTTCAT | Dusp19-B-R | TGCCTCTGCTGGAGTACAGA |
| Psmc3-A-F | GGGTGGAGAGAAGACGTTGA | Psmc3-A-R | TATTCAGTGGGCAGGGTCTC |
| Psmc3-B-F | CCAATGTCATCGAGTTGCTG | Psmc3-B-R | GCGCCATCTCCAATAAACAT |
| Psmc3-C-F | CAGACCAAGGCCACCTTCT | Psmc3-C-R | TCAGACCAGCGGACAGACTA |
| Sgne1-A-F | AGGAGCTATCCAGCACGTCA | Sgne1-A-R | CTTGTTCCACTTGCCCAAAC |
| Sgne1-B-F | TGTCCTCTTGGGAAAACTGC | Sgne1-B-R | CTTGGCCCAAACAGCATAAC |
| Plcb2-A-F | TCTCCAAAGAGGGACACTTGA | Plcb2-A-R | GCTTCACCAGGATCTTGTCC |
| Plcb2-B-F | CAAGGAGAACGTAGGCAAGG | Plcb2-B-R | GAAACCAGACCATCCCCTCT |
| Plcb2-C-F | CGAGACCCTCGACTCAACTC | Plcb2-C-R | TTTTCCAGGGGATCTGTGAG |
| Plcb2-D-F | AAGACCTCCCCGTATCCTGT | Plcb2-D-R | CTTGAGCTCCGTGAAGGAAG |
| Plcb2-E-F | TCCAGCCCACCAAGTTTATC | Plcb2-E-R | GACTCCAATGCTGAGGAGGA |
| Ubr1-A-F | GCCGTCGTAAAAGTGTCGTC | Ubr1-A-R | AGAAGCCCCCTCCAGTAGAA |
| Ubr1-B-F | TGTGTGCTCTGTATGGACTGC | Ubr1-B-R | TTTCTGGTGAGCCATAACCAC |
| Ubr1-C-F | CAGGTGTGTATGCCACTTGC | Ubr1-C-R | TCCCAGTTTGTCCTGGCTAT |
| Ubr1-D-F | TGTCATTACTGAAACGCTGCT | Ubr1-D-R | AAAAGGCACAAATTCATGCAG |
| Ubr1-E-F | AAGCATACACCTGCCACTCTC | Ubr1-E-R | CAGGTAGGTTTCTGGCGATG |
| Ubr1-F-F | TGTACCTGGAGTGGGAAATG | Ubr1-F-R | GCATTCATGGCTGAACTTCC |
| Ubr1-G-F | GGGCTTGCTGGAAGAGAAG | Ubr1-G-R | AGAGGATGCACGTCAGCAC |
| Ubr1-H-F | GGAAGAGAGCACCTCAGCA | Ubr1-H-R | ACCCGATATTCTGGCAAGG |
| Ubr1-I-F | CCGCAGAAGATCAACAGTGA | Ubr1-I-R | CCGACCAGAACATGGAAGAG |
| Ubr1-J-F | GCTCGGCTCCTGTCAGTTAT | Ubr1-J-R | TAAGGCAGGATCTCCACACC |
| Ubr1-K-F | AAATTTGTTCCTGCTTTTCCA | Ubr1-K-R | TCATGATTGTCTTGAGGCAGA |
| Ubr1-L-F | GCAACAGCACTGCATTATAGAAG | Ubr1-L-R | TGTGCAATTCCTCTTTAGACCAT |
| Dusp2-A-F | AGAGGACATCCGGAAGCAG | Dusp2-A-R | TAGGGCAAGATTTCCACAGG |
| Dusp2-B-F | GGCCGAAAATAGCAACTCTG | Dusp2-B-R | AGATGAGGAGGGAGGAGCTG |
| IL1a-A-F | GCTTGAGTCGGCAAAGAAAT | IL1a-A-R | TGTTGCAGGTCATTTAACCAA |
| IL1a-B-F | CGTCAGGCAGAAGTTTGTCA | IL1a-B-R | GCTCCACTAGGGTTTGCTCTT |
| IL1b-A-F | TCTCCAGCCAAGCTTCCTT | IL1b-A-R | TGTCGTTGCTTGGTTCTCC |
| IL1b-B-F | GCTGAAAGCTCTCCACCTCA | IL1b-B-R | AGAGGCAAGGAGGAAACACA |
| Ptpns1-A-F | GGAGGGGCCTTAGTCGTTC | Ptpns1-A-R | GATATTCCGGGGAGAGAAGC |
| Ptpns1-B-F | TACAATCTGGAGGGGGAACA | Ptpns1-B-R | GTTGGTCGTGCTTCACCTG |
| Ptpns1-C-F | GCTCATAGAGAGGACGTGGTG | Ptpns1-C-R | GGCCACTCCATGTAGGACAA |
| Ptpns1-D-F | CAAGCCTGAGCCATCTTTCT | Ptpns1-D-R | TGTTGGCTGAAAACTTGCTG |
| Ptpra-A-F | GTCAGCATGGATTCCTGGTT | Ptpra-A-R | ATGACTCCCAGCTTGCTTGT |
| Ptpra-B-F | CAATTATTGCGGTGATGGTG | Ptpra-B-R | TGTGCAGCGATGAATTTGTT |
| Ptpra-C-F | AGTGCACCTGACACCTGTTG | Ptpra-C-R | ATCCACTTTGCGTTCCGAAT |
| Ptpra-D-F | GGCACCTTTGTTGTCATCG | Ptpra-D-R | CACACTTCTCCTGGCCTCTC |
| Ptpra-E-F | TGGGAGTGGAAGTCCTGTTC | Ptpra-E-R | GGCCTTGTCACCTGTCACTT |
| Ubce7ip5-A-F | GGAAATCAAACCCCAATCCT | Ubce7ip5-A-R | GGGCTACAACAGCAGGAGAG |
| Ubce7ip5-B-F | TTTAGCCCGATGGAAGTCAC | Ubce7ip5-B-R | GGCAGCCTGAAATAGAGTGC |
| Ubce7ip5-C-F | CCCTCATCCTTCCCTGAAA | Ubce7ip5-C-R | GGGAACGTTGCTTCTCACTC |
| Ubce7ip5-D-F | CCTCCTGCAAACAAGCATTT | Ubce7ip5-D-R | TGAGTGACCATGAGGCAGAG |
| Ubce7ip5-E-F | ATGGAGCTTTGTGGATGGAG | Ubce7ip5-E-R | AGACTGCCACACTGCTGATG |
| Plcb1-A-F | AGAGCCTGGTGAATCGAGAG | Plcb1-A-R | GTTGCCAAGCTGAAAACCTC |
| Plcb1-B-F | CTTGGAACAGCGCATGATAA | Plcb1-B-R | GCTGCTGTTGGGCTCATATT |
| Plcb1-C-F | TGAAATCCTTTACCCACCTCTG | Plcb1-C-R | GATTCCAGTGGGTATTTTTCCA |
| Plcb1-D-F | ATCCTCCTTTCCTTTGAGAACC | Plcb1-D-R | TCTACAAATTCCACCGGAGACT |
| Plcb1-E-F | CAGCCTGTCAAGTTTGAGTCC | Plcb1-E-R | TCCCAGACAGGGTTTACAGC |
| Plcb1-F-F | TTGGGACTTATGTGGAAGTGG | Plcb1-F-R | ATTCACCCCATTCTCTGCTG |
| Plcb1-G-F | CCTGGAGGATGAAGAGGAAGT | Plcb1-G-R | AGCTGCTGTTGTTGCTTGTCT |
| Plcb1-H-F | CCCTGGATGCAGAAATGACT | Plcb1-H-R | GGCAGCCTTTTGAACTTGTC |
| Plcb4-A-F | GGATTTGATCGGGTAGACCTC | Plcb4-A-R | CCTGATGCGAAGGTTCTAGTG |
| Plcb4-B-F | CAAACAACGTCAGTCCGATG | Plcb4-B-R | AGGTGTTGTGGGAGGAACTG |
| Plcb4-C-F | TGAAAATGCCCCTGTCTTCT | Plcb4-C-R | TTGTATTCAGGGTGGGCTTC |
| Plcb4-D-F | TGGAAGACGACAATGAAGAGG | Plcb4-D-R | GTCCACAGGGGTTTCAGAGA |
| Plcb4-E-F | ATCATGCGGGTACCTTCTCA | Plcb4-E-R | GTTTCAATGCCCATTGCTCT |
| Plcb4-F-F | TGCCTGATGGATTTGGAGAT | Plcb4-F-R | GTGATCCGTTGTCAGGGTCT |
| Plcb4-G-F | GAAGGCGATGAAGAAGAAGG | Plcb4-G-R | TCCAGCTTCAGTTTGCACTC |
| Pcsk2-A-F | TTTGCATCTTCCCTCTTCGT | Pcsk2-A-R | CCAATGGTCACTCCTTTTCC |
| Pcsk2-B-F | CAAAGCAATGGTACCTGTTCAA | Pcsk2-B-R | CTTGAAGCATAGCCGTCACA |
| Pcsk2-C-F | GGCAAAGGCAGCATCTATGT | Pcsk2-C-R | TGCATTTGTTTTGAGGGTCA |
| Pcsk2-D-F | CCGGAGAGATTCCATTGTGT | Pcsk2-D-R | AACTGTGGAGCAAGGAGAGG |
| Foxa2-A-F | CAGACCACGCGAGTCCTAC | Foxa2-A-R | GTTTGGCGTGTGTGTAGCTG |
| Foxa2-B-F | AACTCGATGAGCCCCATGTA | Foxa2-B-R | GAGGCAGGTGCTCCCTTTAG |
| Foxa2-C-F | GCCTCTCAGGCTCAGCTC | Foxa2-C-R | CCACTCAGCCTCTCATTTCC |
| Foxa2-D-F | CTGGCTGCAGACACTTCCT | Foxa2-D-R | CCTTTCTCCTGGTCCGGTA |
| Sstr4-A-F | CCCAGCCTCTTGGTCCTT | Sstr4-A-R | CCTGGTGTCAGCGAAGACT |
| Sstr4-B-F | GACCGCTATGTGGCTGTTGT | Sstr4-B-R | TCTTCCTCAGCACCTCCAGT |
| Sstr4-C-F | CTGTGCCAATCCCATTCTCT | Sstr4-C-R | GGGCTCTTTCAAGCAGTCAC |
| Bcl2l1-A-F | TCGCTAAACACAGAGCAGACC | Bcl2l1-A-R | TCTCCTTGTCTACGCTTTCCA |
| Bcl2l1-B-F | TTCGGGATGGAGTAAACTGG | Bcl2l1-B-R | TGGCTTTCACAGAAGTGTGG |
| Bcl2l1-C-F | CCATTGCTACCAGGAGAACC | Bcl2l1-C-R | AGCAAGGTGGACTTTCAGCA |
| Scand1-A-F | CGGACTGCGAAACAAAGG | Scand1-A-R | TCAGGGGTAGGGTGTCAGTC |
| GHRH-P1 | CAGTGGGACCTGAGCAGAAC | GHRH-P2 | GATGAGAATGGGGGTTTTATTG |
| Src-A-F | CCAAGACCCCGGATACTACA | Src-A-R | GTACCACTCCTCAGCCTGGA |
| Src-B-F | ACACGAGGAAGGTGGATGTC | Src-B-R | GTGCCTGGCTTCAGAGTTTT |
| Src-C-F | GCTTCGGAGAGGTGTGGAT | Src-C-R | TCAGCAGAATCCCAAAGGAC |
| Src-D-F | GCTCTGTACGGCAGGTTCAC | Src-D-R | CACTGTACCAGCCTCCACCT |
| Src-E-F | CCTTTTGGAGCATGGAAGG | Src-E-R | TAAACTGCCCTTGGTCTTGG |
| Plcg1-A-F | TGACCGCTACCAAGAAGACC | Plcg1-A-R | CAAGGAGGAACTGCTGGAAC |
| Plcg1-B-F | AACGCTTTGAGGACTGGAGA | Plcg1-B-R | CCTCGATGGACAGGATGACT |
| Plcg1-C-F | GCACACTCTCACCACCAAGA | Plcg1-C-R | GAGCCCCAGTCTCTATGCAG |
| Plcg1-D-F | CTCGAGTGAGAAGTGGTTCC | Plcg1-D-R | CTGGCCTTCCTGCTGTACTC |
| Plcg1-E-F | ACATATGCTGATGCGAGTGC | Plcg1-E-R | AGTGGGCTGTTCTCATCCAG |
| Plcg1-F-F | TGTGGAAAAGCAAGATGGTG | Plcg1-F-R | TTAGGGTAGATGCGCGAGAG |
| Plcg1-G-F | GGCACAGAACGTGCTTGTTA | Plcg1-G-R | TACACCACAAAGCGCAGAAA |
| Plcg1-H-F | AGTGGACAACGGACTGAACC | Plcg1-H-R | CAGTTCATGGCATTCCACAG |
| Plcg1-I-F | CCCTTGTGCTGTGTCTTCCT | Plcg1-I-R | CCCTTGTGCTGTGTCTTCCT |
| Hnf4-A-F | GGGGAGAATGCGACTCTCTA | Hnf4-A-R | GAGAGGTGATCTGTTGGGACA |
| Hnf4-B-F | GGTCAAGCTACGAGGACAGC | Hnf4-B-R | TACTGCCGGTCGTTGATGTA |
| Hnf4-C-F | TTTGATCCAGATGCCAAGG | Hnf4-C-R | GAGGGTATGAGCCAGCAGAA |
| Hnf4-D-F | CATCGTCAAGCCTCCCTCT | Hnf4-D-R | TGACCCAGCCTCAGAAAGTT |
| Mmp9-A-F | AGGCAGCGTTAGCCAGAAG | Mmp9-A-R | CGACACCAAACTGGATGACA |
| Mmp9-B-F | AGACTTGCCGCGAGACAT | Mmp9-B-R | AGTGGTGCAGGCAGAGTAGG |
| Mmp9-C-F | AAGGCAAACCCTGTGTGTTC | Mmp9-C-R | TGTGGTTCAGTTGTGGTGGT |
| Mmp9-D-F | AGACGACATAGACGGCATCC | Mmp9-D-R | CTGACGTGGGTTACCTCTGG |
| Mmp9-E-F | GCAGACCAAGAGGGTTTTCTT | Mmp9-E-R | CTCCACTCCTTCCCAGTCTCT |
| Mmp9-F-F | CCGTGCAGTGCAAGTCTCTA | Mmp9-F-R | GTGGATAGCTCGGTGGTGTT |
| Onecut1-A-F | GCCGATGTGAAGACTGGACT | Onecut1-A-R | TCATCCCGCATAAGTGTGAA |
| Onecut1-B-F | GTTCCCTCACCATCATCACC | Onecut1-B-R | ATCTCTTCCATCTGCCCTGA |
| Onecut1-C-F | GCAGGTCAGCAATGGAAGTA | Onecut1-C-R | GCCACTTGTCCAGACTCCTC |
| Csk-A-F | ACAGATCGGCCTTCCTGTAG | Csk-A-R | GCCACTGCGGTAGAACTCAT |
| Csk-B-F | CTACACCACAGATGCCGATG | Csk-B-R | AGCTTGCCTGTGTCCTGAGT |
| Csk-C-F | AAGACAACGTGGCCAAAGTC | Csk-C-R | GGTGGGTCCATGAGAGAAGA |
| Csk-D-F | GGCCTGAACTTGAACTGAGC | Csk-D-R | GGTGTCCTCATGGAGTGGTT |
| Map2k1-A-F | CTGAGTTGCAGGCTCTTTCC | Map2k1-A-R | CTTGTGCTTCTCCCGAAGAT |
| Map2k1-B-F | GCTGGAAGAATTCCTGAGCA | Map2k1-B-R | TTTGGAGGAGGCTCATTGAC |
| Map2k1-C-F | GGCAATTTTTGAGTTGTTGGA | Map2k1-C-R | ATACAGGCAGCCAGCTAGTGA |
| Map2k1-D-F | CTAGTGACCTGGGTGGTCGT | Map2k1-D-R | AAAGTGGTTAAAAGGGGTTTCA |
| Pias1-A-F | GACGCAAGATGGCGGACAGT | Pias1-A-R | CACCTGTTGTGGTGTCAAGG |
| Pias1-B-F | GCCCACCAGTCTAGCTTCAG | Pias1-B-R | GGGATTGTCAGTCGCATTTT |
| Pias1-C-F | ACTACCAGCCTACGGGTTTC | Pias1-C-R | CGAGGCTTGATGAGGAAGAC |
| Pias1-D-F | TCCCTGTCTCCTACGTCACC | Pias1-D-R | GCATAGAGTTCTCTTCTGCCAAG |
| Cbl-A-F | GAGAGGCCCCTCCTTCTC | Cbl-A-R | TAGGCTGGGAATTCTCCTCA |
| Cbl-B-F | AGATGGAGACGCTTGGAGAA | Cbl-B-R | ACAGCTCAGCCGAAAGATGT |
| Cbl-C-F | CACCCTGGTTACATGGCTTT | Cbl-C-R | CCATGGAGAATGGAGAGGAA |
| Cbl-D-F | CCCCAAATTACGACGATGAT | Cbl-D-R | GCCTAGGGACATCTGCTCTG |
| Cbl-E-F | TGATCCTTGGAATGGGAGAG | Cbl-E-R | TCATCCTCGTTTTCGGATTC |
| Cbl-F-F | CCCAAGCACTGTCTGTAGCA | Cbl-F-R | AGCCACTGCTTTCCTAGGTG |
| Tyk2-A-F | CTGCCTGAGGTCACACAGAA | Tyk2-A-R | AAACGAGGCAGAGTCCAAGA |
| Tyk2-B-F | AGGGGCAGAGACTTCCTCAG | Tyk2-B-R | GGTGCCTGTCACCAGTACCT |
| Tyk2-C-F | GACCCCTGCTACATCCAGAA | Tyk2-C-R | CACAGCTTGGCCTGTACAAA |
| Tyk2-D-F | GATGGCTATTTCCGCTTGAC | Tyk2-D-R | GGACAGCTGGGTGATTTCAT |
| Tyk2-E-F | GGCCTCTCAACCTCAGTCAG | Tyk2-E-R | TAGCTTGATGAAGGGGTTGG |
| Tyk2-F-F | GAATCTGGTTCACGGGAATG | Tyk2-F-R | GTCATTGGTTGGGTCGTAGC |
| Tyk2-G-F | ACCCCACTGTTTTCCACAAG | Tyk2-G-R | CTGGGGCATACCAGAACACT |
| Tyk2-H-F | CCTAGCCAAGGCTGTACCTG | Tyk2-H-R | AATCCCAATTAGCCCCTTTG |
| Tyk2-I-F | GCCATGACCTGAACAGACCT | Tyk2-I-R | GGGGCCCTGCTCTACTATTT |
| Ptpn9-A-F | GACCGAGAGCTGCTGAGG | Ptpn9-A-R | GGCATAATTAGACCCGCACA |
| Ptpn9-B-F | GTGGTGCTCCAAGCTCTGTT | Ptpn9-B-R | TGGAGACATGGAACAGTGGA |
| Ptpn9-C-F | CGGGGCATCTATGAGGAGTA | Ptpn9-C-R | GCTGAGGAAGGGACACCATA |
| Ptpn9-D-F | ACGCCAGGTTACCCACTTC | Ptpn9-D-R | GGCCCTTTCTAGTGGCAAGT |
| Ptpn9-E-F | CAGCCTTTCCCTTTGGCTA | Ptpn9-E-R | GGGTCCAAGAAGCAAGGAA |
| Ptpn9-F-F | TCTTTGTAGAAGTCTCTGGGGATT | Ptpn9-F-R | GGAGAGCTGGGCATTCAGTA |
| Slc2a4-A-F | CAGCGCCTGAGTCTTTTCTT | Slc2a4-A-R | ACCAGAATGCCAATGACGAT |
| Slc2a4-B-F | TCATTCTTGGACGGTTCCTC | Slc2a4-B-R | CGTGAAGACCGTATTGACCA |
| Slc2a4-C-F | CATAGGAGCTGGTGTGGTCA | Slc2a4-C-R | CTGTTTTGCCCCTCAGTCAT |
| Slc2a4-D-F | GCAGGAGGTGAAACCCAGTA | Slc2a4-D-R | TTGGGAGGGTGTATCCTTTG |
| Tcf2-A-F | CCTCAACCCCTTCTTTTTCC | Tcf2-A-R | CGTTGCTTTCTGACGTACCA |
| Tcf2-B-F | CCCATGAAGACCCAGAAGAG | Tcf2-B-R | TGTAGCGCACTCCTGACATC |
| Tcf2-C-F | ACCAGACGCACAACCTGAAC | Tcf2-C-R | GGGGTTCCTGCTTATGTGC |
| Tcf2-D-F | GCGGTGACTCAGCTACAGAA | Tcf2-D-R | ACCCTCCTTGGTCCTCATCT |
| Ccl9-A-F | CAAAGGAGGGCATTATGAGC | Ccl9-A-R | TTAGGTGGTCACTGGGGAAG |
| Ccl9-B-F | TCACAACCACGGACCTACAA | Ccl9-B-R | CCAAAGTCCCACAGACCACT |
| Crk-A-F | CTGCTGGGAGGCTGACAC | Crk-A-R | TCCTCCTGCCTGAGAATCAC |
| Crk-B-F | TCATTGCCTGCTTTACTGGA | Crk-B-R | ATGGGAAGTGACCTCGTTTG |
| Crk-C-F | CAGAAGCGAGTCCCTAATGC | Crk-C-R | AACAGTCTGCTGCCACTTGA |
| Crk-D-F | AACCTGTGGAAAGCTGATGG | Crk-D-R | GATTGGTCCTTCCACTCCAA |
| Crk-E-F | CACACTGCTACCACAGCTCAA | Crk-E-R | AGAGACCAGCAAGTGCCTTT |
| Calm2-A-F | GAGTGGAGCGAGCGAGTC | Calm2-A-R | TCACTTCGCTGTCATCATTTG |
| Calm2-B-F | TTCGCCATGTGATGACAAAC | Calm2-B-R | CCACAGTCCACGCAGAGTTA |
| Vav-A-F | GGTGTTGTAGTTGTCCCCACT | Vav-A-R | GTCTTCCTCTGCGGTGTCAT |
| Vav-B-F | CCTGTCTGCTCTGTCATGGA | Vav-B-R | CTGACTCCACCTGACTGCAA |
| Vav-C-F | TGGTTTATGGCCGTTATTGC | Vav-C-R | CGAGGCTTTGAGGTCGTAAG |
| Vav-D-F | TGGACAAAGCACTGCTCATC | Vav-D-R | ACCAGTTGTGCTCAGCCTCT |
| Vav-E-F | TGGGTCTGCCTAAGATGGAA | Vav-E-R | GTGTCCAACGACTTGAAGCA |
| Vav-F-F | CCGGATCACAGAGAAGAAGG | Vav-F-R | CTTTCGTGGAGGACTGGAGA |
| Socs5-A-F | GCGTAGTGGGAGCTTACTCG | Socs5-A-R | AGAAACGCTGTCCATGTCCT |
| Socs5-B-F | CTGTTCCACAAAGACCCAGAG | Socs5-B-R | TGGGTTGACCTGTGCAGTAG |
| Socs5-C-F | CCCAACGCACAAATACACAC | Socs5-C-R | CGTGACAGTGGAGGAGTGAA |
| Socs5-D-F | GCAGTGGAACCACAACTTCA | Socs5-D-R | CATAGCAAATGCCCCAGAGT |
| Socs5-E-F | AAGATGGCTTGCAGGTGTTC | Socs5-E-R | GAGAAAAACACCCCACTGGA |
| Socs5-F-F | GTAAAAGCCCCAGTGTGCAT | Socs5-F-R | ACAGAACCGGGAACACAGTC |
| Socs5-G-F | TTGCTTCTCGTGTTGGACTG | Socs5-G-R | TCACTTCTCTGGGTCCTGCT |
| Sos1-A-F | TGTTCTAGTTGGGTGCAAGG | Sos1-A-R | TCAGCACACATTGCCACTTT |
| Sos1-B-F | AAAGCTCGTGGGGAATTATG | Sos1-B-R | CTTTGAAGCCTTCGCCTATG |
| Sos1-C-F | CTGGGGCAGCACTTTATTTG | Sos1-C-R | GCTAGCACCAGGGAGTCTTG |
| Sos1-D-F | AGCCAAACACGAGAGACACA | Sos1-D-R | TGGATCTGCGTACATGTGGT |
| Sos1-E-F | AAGCTGGGATCCCCATTATC | Sos1-E-R | CTGCACAGCCCGGTATAGAT |
| Sos1-F-F | ATTGCTCGGCAACTCACTTT | Sos1-F-R | CCACTCTCCTCCTCTTGCTG |
| Sos1-G-F | TGAGGAGACACGGGAAAGAG | Sos1-G-R | CTTCATCAGTGCCCTTGGAT |
| Sos1-H-F | GCAAGCCCTTTTCATTCAAG | Sos1-H-R | ATTCTCCAGCAGTGGTGGTC |
| Sos1-I-F | CCCAAACTCCCTCCAAAAAC | Sos1-I-R | GTACAGTGCTGGGCCTTGAT |
| Sos1-J-F | GGCTGTGGTACCTGACCTTG | Sos1-J-R | TGCCAGCCAAACAAAGTAGT |
| Sos1-K-F | TTGCATTGCCTAAATCATGTG | Sos1-K-R | TGATGGCTGTCAAAGAATGG |
| Cul5-A-F | CAAGTCTCGCCCCGTCTC | Cul5-A-R | TTGCACTGTCCTGGAGTCTG |
| Cul5-B-F | GCTCATGCTTGATACGTGGA | Cul5-B-R | ATGCTCCTCCAAGTCCTTCA |
| Cul5-C-F | GCATGATCAAGCGAAATGAA | Cul5-C-R | CGTCGGGTAAGATGAGCTTT |
| Cul5-D-F | GGAAAACGCCATTAAGCAAA | Cul5-D-R | ACACAGCCAACTGAAACGTG |
| Cul5-E-F | CACTGCCACCATCTCATGTC | Cul5-E-R | TCCGGATGTACCTGTGTTCA |
| Cul5-F-F | CATGTTCCTGCCTCAGAAGA | Cul5-F-R | ATCCCCAGCATTTTTCAGTG |
